# Organizational and behavioral models in the management of patients with developmental and epileptic encephalopathy, Lennox-Gastaut syndrome and Dravet syndrome in Italy: a focus on the transition from pediatric to adult care

**DOI:** 10.3389/frhs.2025.1632564

**Published:** 2025-11-07

**Authors:** Carlo Di Bonaventura, Antonietta Coppola, Giancarlo Di Gennaro, Lucio Corsaro, Emanuele Corsaro, Lorena Trivellato, Gianluca Vaccaro

**Affiliations:** 1 Policlinico Umberto I, Sapienza University of Rome, Rome, Italy; 2 Epilepsy Center, University Hospital Federico II, Naples, Italy; 3 IRCCS NEUROMED, Pozzilli (IS), Italy; 4 Director of the Department of Behavioral, Pharmacoeconomic, and Predictive Research & Consulting, BHAVE, Rome, Italy; 5 Health, Digital and Marketing Research, BHAVE, Rome, Italy; 6 Department of Qualitative Research, BHAVE, Rome, Italy; 7 Methodological Advisor – BHAVE, Rome, Italy; 8 Sociologist UO Education and Health Promotion, Asp Catania, Catania, Italy

**Keywords:** Developmental and Epileptic Encephalopathies (DEEs), Lennox-Gastaut syndrome (LGS), Dravet syndrome, transition, patient journey, rare disease, unmet needs, drug-resistant epilepsy

## Abstract

**Introduction:**

Developmental and Epileptic Encephalopathies (DEEs) are rare and complex conditions characterized by drug-resistant seizures and severe neurocognitive impairments. Management models for these disorders are often inconsistent, and the transition of care from pediatric to adult services represents a critical phase. This transition is frequently managed in an unstructured manner, leading to significant consequences for care continuity and the quality of life of both patients and their families.

**Methods:**

A cross-sectional observational survey was conducted among specialists (neurologists and pediatric neuropsychiatrists) and caregivers of patients with DEEs, particularly those diagnosed with Lennox-Gastaut Syndrome (LGS). The aim was to analyze organizational models, transition pathways, and patient and caregiver experiences, identifying existing gaps in care and comparing these models with those used for another DEE, Dravet Syndrome (DS).

**Results:**

The survey involved 47 physicians and 30 caregivers. Findings revealed substantial fragmentation in management models and the absence of standardized transition pathways in 54% of respondents. The transition of LGS patients to adult care centers is often left to individual families, with a dropout rate of 40% for LGS—similar to that observed in DS patients (38%). Caregivers reported stress, organizational difficulties, and a perceived decline in the quality of adult care. Furthermore, 53% of caregivers stated they received no support services following diagnosis.

**Discussion:**

The lack of standardization in transition pathways represents a critical barrier to ensuring continuity of care for DEE and LGS patients. Developing structured, best-practice—based transition models, enhancing caregiver support, and fostering a multidisciplinary approach are essential to improve quality of life and ensure effective disease management into adulthood.

## 1 Introduction

Developmental and Epileptic Encephalopathies (DEEs) are a heterogeneous group of complex neurological conditions that can have both genetic and non-genetic etiologies. Most of these disorders are genetic in origin and manifest in early childhood (1–3). Genetic DEEs have been associated with mutations in numerous genes involved in functions such as neuronal migration, proliferation, and organization, neuronal excitability, synaptic transmission, plasticity and metabolic pathways (4, 5). Clinically, these encephalopathies are characterized by a combination of drug-resistant epileptic seizures and neurodevelopmental disorders such as intellectual disability, behavioral, psychiatric, autonomic and motor impairments. Epileptic activity itself can further impair neurodevelopment, thus worsening the underlying condition.

A paradigmatic example of these conditions is Lennox-Gastaut Syndrome (LGS), a rare and severe form of childhood epilepsy that develops before the age of 18 with a peak at 5 years. The syndrome is characterized by the occurrence of various types of seizures in the same patient, associated with cognitive deficits and distinctive electroencephalographic abnormalities. Children with LGS have a higher risk of mortality than the general population, and the complexity of the syndrome negatively affects not only intellectual development but also academic abilities and social life (6).

Beyond the direct clinical manifestations, DEEs have a devastating impact on quality of life that extends beyond seizure control, involving psychological, social and economic aspects. The caregiver burden is substantial, with caregivers experiencing significantly elevated levels of stress, anxiety and depression (7, 8): studies report a history of treatment for clinical depression and post-traumatic stress disorders in caregivers of patients with DS and LGS. Also, physical burden is substantial: chronic exhaustion and physical stress result from the constant vigilance required to manage uncontrolled seizures and developmental disabilities, further exacerbated by sleep deprivation caused by nocturnal seizures (8, 9). The economic impact is equally significant, with important direct and indirect costs per person. This substantial economic burden frequently translates into reduced work productivity, deteriorating social relationships, and compromised economic stability, ultimately threatening quality of life and the stability of the family unit (8).

Despite this substantial burden, few effective treatments exist for the multiple seizures and associated comorbidities, and most patients have an unfavorable long-term prognosis. Due to the complexity of the disorder, few randomized trials have been conducted on LGS, and some commonly used treatments are not supported by strong scientific evidence (10).

Given the multifaceted nature of these conditions, DEEs require complex lifelong multidisciplinary care approaches. Multidisciplinary teams should ideally include specialized neurologists, child neuropsychiatrists, psychologists, rehabilitation specialists, geneticists, dietitians, specialized nurses and care coordinators. Optimal management requires a balance between highly specialized services provided in centers of excellence and more accessible local services. However, clinical reality often shows significant gaps in the implementation of these ideal models. Within this comprehensive care framework, the transition of DEEs patients from pediatric to adult care often represents a critical phase due to the complexity of these conditions (11).

As highlighted in recent studies (11, 12), the pediatric care model for DEEs involves a multidisciplinary approach and significant parental involvement, addressing not only seizure control but also a wide range of related needs, such as intellectual disabilities, psychiatric disorders, and socio-educational difficulties. This challenge is compounded by limited awareness of therapeutic approaches in adult care settings and can have a significant impact on the quality of care and patient health if not properly managed. The lack of structured pathways is evident, and dropout rates can be high following transition. Therefore, the transition to adult care centers must be carefully planned through close collaboration between patients, families, pediatric and adult medical teams to ensure continuity of care and prevent patient dropout.

Based on these premises, the present study aimed to examine the organizational models for the management of patients with DEEs, with a particular focus on LGS, and to compare them with the organizational models for the management of patients with Dravet Syndrome (DS). The survey identified gaps in current healthcare, highlighting fragmentation and inconsistency in management models, as well as best practices that can serve as models to improve the overall care provided to such patients and their quality of life.

## 2 Materials and methods

### 2.1 Sample

A cross-sectional observational sociological study was conducted in Italy between December 2023 and May 2024, involving specialist neurologists, pediatric neuropsychiatrists with expertise in DEEs (LGS and DS), and caregivers of patients affected by DEEs/LGS and DS.

The survey was carried out using standardized questionnaire-based interviews. Specifically, two questionnaires were used: one administered to a sample of 47 physicians (37 neurologists/neuropsychiatrists for DEEs/LGS and 10 specializing in DS), distributed by geographical area, and another to 30 caregivers, also geographically distributed and exclusively of DEEs/LGS patients, also geographically distributed. Caregivers included both those of patients with DEEs/LGS (*n* = 12) and those of patients with Dravet Syndrome (DS; *n* = 18). The complete questionnaires used in this study are available as supplementary materials.

Participants were recruited randomly through internal databases from Bhave, with a small proportion (less than 5% of respondents) recruited through reference clinicians using snowball sampling.

### 2.2 Screening criteria

For physicians:

- Specialization in neurology, pediatric neuropsychiatry, or neuropediatrics (hospital pediatrician)
- Specific expertise in treating DEEs in general, or LGS or DS
- Experience treating at least one patient with LGS

For caregivers:

- Being the caregiver for a patient with DEEs or LGS
- Providing care for at least one year
- Caregivers categorized by patient age group (Categories: 0–14; 15–17; 18–29; Over 30)
- Caregivers of DS patients were also included, recruited with the same methodology and applying the same eligibility criteria, to allow comparative analysis.

### 2.3 Objectives

The project was structured into two main phases with the following specific objectives for each phase:

1. Quantitative Research—Organizational Models (Physicians and Healthcare Facilities): This first phase aimed to identify, define, and quantify the main organizational models for the management of DEEs patients, particularly those with LGS. This phase sought to compare emerging healthcare and social care organizational models with those related to other rare DEEs, such as DS.
2. Quantitative Research—Functional Journey (Caregivers): This second phase focused on reconstructing and defining the patient's journey through the experiences reported by caregivers, from the onset of symptoms to diagnosis and long-term disease management. The objective was comparative in nature, meaning it aimed to contrast the experiences emerging from the direct accounts of caregivers with the pathways reported by healthcare facilities and physicians to identify the main effective models of care organization, define turning points in the care pathway and their functional, emotional, and social implications, and explore barriers affecting patient pathways and unmet needs in terms of care received at different stages.

### 2.4 Methodology

The entire research process was conducted in compliance with the ethical guidelines and codes of conduct of EphMRA (European Pharmaceutical Market Research Association), ASSIRM (Italian Market Research Association), ICC, ESOMAR, and Farmindustria. Questionnaires were administered in compliance with Italian privacy laws, pharmacovigilance regulations, and the Italian Communications Authority (AGCOM). Data were processed anonymously and confidentially to ensure privacy protection and compliance with personal data protection regulations (EU Regulation 2016/679—GDPR). The study was also conducted in accordance with the principles of the Declaration of Helsinki.

The questionnaires were specifically developed and validated by a multidisciplinary board of research methodologists, neurologists, and neuropsychiatrists. The design followed key methodological criteria:

- Intersubjectivity: ensuring evaluations were independent of the interviewer's subjective judgment.
- Standardization: maintaining uniformity and reproducibility of results across repeated trials.
- Sensitivity: using semi-structured multiple-choice questions, Likert rating scales for attitudes, and numerical or ranking scales to capture the most appropriate range of evaluation scores.
- Reliability: assessed through test-retest procedures, ensuring stability of results over time.
- Comprehensibility: verified in a pilot phase (5% of the total sample), ensuring that the questionnaire effectively captured the information required to meet the study objectives.

The methodological framework was grounded in Outcome Research, integrating clinical and sociological perspectives to evaluate both clinical outcomes and subjectively perceived outcomes through established observational approaches (13). Recent developments in health services research emphasize the importance of patient journey analysis as a paradigm to capture the full experiential pathway of patients across healthcare systems, from first symptoms to long-term management. This approach enables the identification of critical points of discontinuity in care pathways, providing a comprehensive view of organizational strengths and weaknesses (14, 15).

The study aimed to reconstruct the Patient Journey of individuals with DEEs/LGS and DS. This model integrated sociological and anthropological perspectives, examining care processes at three levels:

- Micro-level: individual experiences and illness narratives (16, 17), focusing on how patients and caregivers assign meaning to symptoms, diagnosis, treatments, and interactions with professionals.
- Meso-level: organizational and professional practices, including therapeutic choices, monitoring, and coordination among healthcare providers.
- Macro-level: institutional and structural factors such as policies, social inequalities, and geographic disparities that shape access to care.

Within this framework, the Patient Journey was articulated into five phases:

1. Pre-diagnosis—initial manifestations and interpretations of abnormal events within the patient's social and cultural network.
2. Diagnosis—the process of naming the condition and the patient's immediate information-seeking activities, shaped by communication methods and access to healthcare facilities.
3. Therapeutic choice—negotiation between physician and patient/caregiver, including evaluation of alignment with personal priorities and systemic constraints (availability of therapies, relocation for treatment, bureaucratic burdens).
4. Living with the condition—long-term adaptation of care pathways, changes in facilities, therapeutic approaches, onset of comorbidities, and patterns of adherence to follow-up and monitoring.
5. Self-evaluation—a transversal process through which patients, caregivers, and healthcare professionals continuously re-assess the illness experience and recalibrate their health perceptions.

In addition, five cross-sectional dimensions intersected these phases:

1. Social dimension: the impact of the disease on the patient's family, work, and social network, and on societal representations of the illness.
2. Psychological dimension: emotional and cognitive consequences, well-being, and support systems available for patients and caregivers.
3. Cultural dimension: interpretations of illness, differences in worldviews, and the coexistence of biomedical and alternative care paths.
4. Political-institutional dimension: recognition and representation of the disease, institutional awareness initiatives, and regional disparities.
5. Administrative and bureaucratic dimension: access to laws, exemptions, waiting lists, and procedures governing continuity of care.

The integration of this model with data-driven approaches highlights the potential of patient journey mapping not only for descriptive and comparative analysis but also for optimizing healthcare services through the collection and processing of patient behavior data. Studies adopting digital and process-mining techniques have demonstrated how patient journey data can reveal critical bottlenecks, resource allocation issues, and efficiency gaps in healthcare systems (18). This integrated perspective reinforces the value of the patient journey as both a conceptual framework and a practical tool for improving patient care and system performance.

### 2.5 Statistical analysis

All quantitative data were analyzed using IBM SPSS Statistics v.29 (IBM Corp., Armonk, NY, USA). Descriptive statistics (frequencies and percentages) were reported for categorical variables, while means and standard deviations were calculated for continuous variables. Differences between groups (e.g., LGS vs. DS, physicians vs. caregivers) were tested using Chi-square (*χ*^2^) tests or Fisher's exact test for categorical variables, and independent-samples *t*-tests for continuous variables. When comparing more than two groups, ANOVA was used, followed by Bonferroni *post-hoc* tests. A significance level of *p* < 0.05 was considered statistically significant. For proportions, 95% confidence intervals (CIs) were calculated. To estimate the magnitude of differences, effect sizes were reported (Cohen's *d* for means, Cramer's *V* for proportions).

### 2.6 Data collection procedure

Data were collected through standardized Computer-Assisted Web Interviewing (CAWI), Computer-Assisted Telephone Interviewing (CATI), and Face-to-Face (F2F) methods. The average interview duration was approximately 20 min for physicians and 25 min for caregivers. Questionnaires were developed by a multidisciplinary board of methodologists, neurologists, and neuropsychiatrists, and were organized into four main sections: (A) respondent profile, (B) organizational models and procedures, (C) patient journey reconstruction, and (D) transition to adulthood.

## 3 Results

### 3.1 Sample structure

A total of 77 respondents participated in the study: 47 physicians and 30 caregivers. Among the caregivers, 18 were relatives of individuals with DS and 12 were relatives of individuals with DEEs/LGS. This distribution was considered in all subsequent analyses, and percentages are reported together with absolute numbers (n/N) to ensure transparency. When interpreting differences between DS and DEEs/LGS caregivers, results should be read with caution given the smaller sample sizes of the subgroups.

The caregiver sample consists primarily of parents of patients; however, siblings of affected patients also represent a portion (12% of the sample). To ensure comprehensive analysis, caregivers of patients of different ages were included, providing a complete picture of the journey and focusing on a particularly critical phase: the transition from pediatric to adult care. The most represented caregiver group corresponds to patients aged 18–29, with a significant portion also caring for minors (24%) and patients over 30 (12%).

The majority of caregivers interviewed are homemakers or part-time employees, with 83% reporting that their current employment status is influenced by their role as a caregiver for a patient with a rare disease. To facilitate interpretation, results were structured to distinguish between physicians' and caregivers' responses, with explicit comparisons across syndromes (DS vs. DEEs/LGS) and between respondent groups where relevant.

Among the responding physicians, 78% are specialists in Neurology, 19% in Pediatric Neuropsychiatry, and only 3% in Pediatrics. A key distinction emerges based on the specific area of expertise: 76% of those treating DEEs/LGS are neurologists, whereas an overwhelming majority of specialists managing DS patients are pediatric neuropsychiatrists (83% of the sample), as this reflects the age at which the diagnosis is definitively made.

Looking more closely at the characteristics of the healthcare facilities where the surveyed physicians practice, distribution across Italy is uneven, with most facilities concentrated in Northern and Central Italy. In addition to hospitals, which account for 24% of participating institutions, the largest proportion of respondents are from university-affiliated healthcare centers (43%).

### 3.2 Patient management

This section first reports physicians' responses regarding team composition and organizational practices, followed by caregivers' perspectives on the availability of support services. Comparative analyses are presented both between syndromes (DS vs. DEEs/LGS) and between respondent groups (physicians vs. caregivers).

The distribution of patients among the centers surveyed showed a significant percentage of patients coming from outside their region of residence: 24% for pure LGS, 27% for LGS-like cases, and 16% for other DEEs. In the case of pure and LGS-like cases, most patients came from central and southern regions, particularly Calabria, Campania, Abruzzo, and Puglia. However, the regional distribution shifts when looking at out-of-region patients with other DEEs, who are reported by specialists to mainly come from Basilicata, Molise, and Calabria.

**Figure 1:**
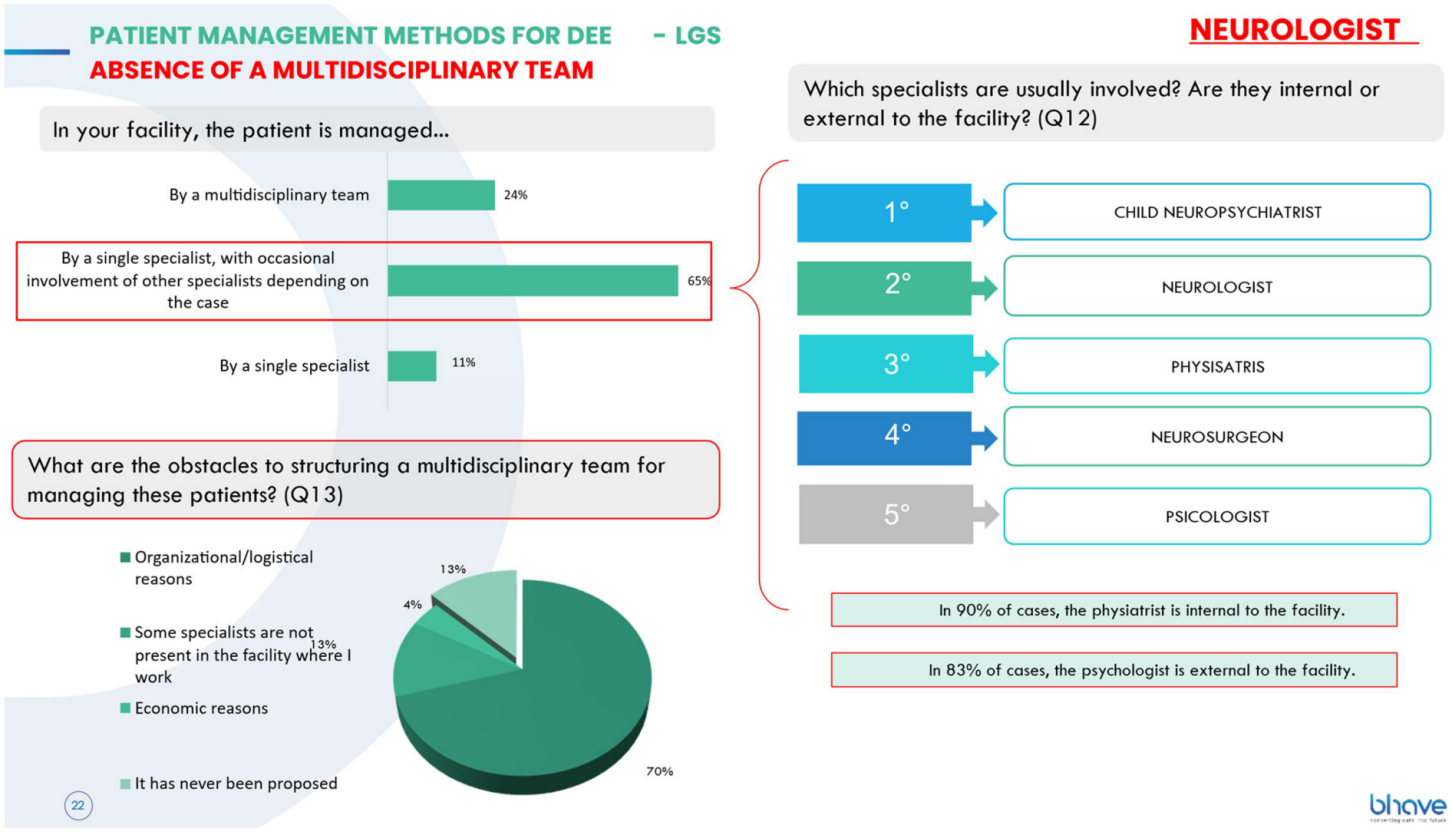
Management of DEEs and LGS patients without a multidisciplinary team: main obstacles include organizational/logistical hurdles, specialist absence, and lack of initiative to propose team creation.

Despite the complexity of these conditions and the urgent need for an integrated multidisciplinary approach, patient management remains predominantly entrusted to a single specialist (65% of cases), with occasional involvement of other professionals such as physiatrists, hospital pediatricians, and psychologists, the latter almost always external to the facility. The main obstacles to the creation of multidisciplinary teams are reported to be organizational and logistical challenges, as well as the absence of certain specialists within the facility or the lack of initiative to propose their creation (Figure 1).

Where available, the team consists primarily of a neurologist—who, in most cases, is responsible for therapeutic decisions—a pediatric neuropsychiatrist, a physiatrist, a psychologist, and a neurosurgeon, with the latter almost always being an in-house specialist. A significant proportion of teams (27%) do not hold multidisciplinary meetings, while those that do have a mean interval of 22 days between meetings, which are predominantly conducted in person and, in some cases, online. From a *social dimension*, the absence of regular multidisciplinary meetings in more than one-fourth of DEEs/LGS centers reveals a structural gap in coordination, with potential consequences on patients' and families' social support networks. However, this difference compared to DS teams (18%) did not reach statistical significance [*χ*^2^(1) = 0.96, *p* = 0.33].

Regarding the services available at the centers for patients and their families, the findings highlight the presence of psychological support services for patients and, in some cases, for their families. In addition to psychological support for patients who cannot easily travel to the center or are cared for at home, 69% of facilities provide training programs on disease management and therapies, while 22% offer remote monitoring systems. However, patient support programs offered by private companies are completely absent.

From a *psychological dimension*, these findings highlight the limited integration of emotional support services for DEEs/LGS families, despite the high burden of care. The stronger presence of training initiatives in these centers reflects a partial but important response to caregivers' need for empowerment.

Psychological support services were more frequently available for DS families (12/18; 66%) than for DEEs/LGS families (5/12; 43%), a difference that reached statistical significance [*χ*^2^(1) = 4.02, *p* = 0.045; 95% CI difference: 1.2%–44.1%]. Conversely, caregiver training programs were significantly more frequent in DEEs/LGS centers (8/12; 69%) than in DS centers (3/18; 17%) [*χ*^2^(1) = 18.21, *p* < 0.001; Cramer's *V* = 0.41, large effect size].

### 3.3 Transition to adulthood

In this section, results are presented separately for physicians and caregivers, and comparisons between DS and DEEs/LGS are reported where applicable.

Regarding the transition to adult care centers, caregivers of DEEs/LGS patients report that this process begins at age 18 in 29% of cases. The remaining respondents describe highly variable situations, reflecting the lack of structured procedures: in 14% of cases, the transition occurs after the patient turns 20 or 25; in 7%, it occurs only when necessary; and in another 14%, the process is left entirely to the family, which has to find a suitable center on its own. A significant proportion of respondents do not know when the transition will occur, partly due to a lack of information, but also because they are caregivers of patients who have not yet reached the transition age. Among families currently being cared for by a pediatric center, 75% report that the topic is not discussed, primarily because the caregivers themselves prefer that their loved ones continue to receive care from the same facility.

**Figure 2:**
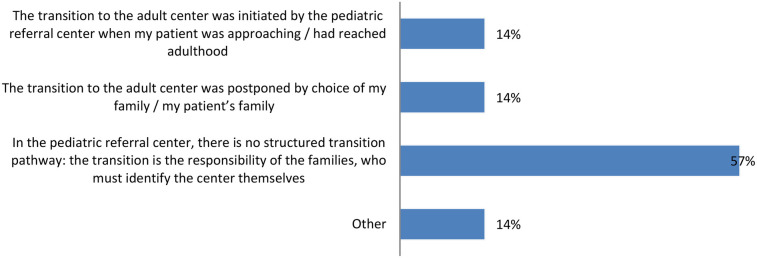
Management of the transition to adult care: 57% of caregivers handled the entire transition process alone due to the lack of a structured transition pathway at the pediatric center.

Among those who have transitioned, a concerning 57% of caregivers state that the entire process was their responsibility, as the pediatric reference center did not have a structured transition pathway, leaving families to find an adult center on their own (Figure 2).

When a pathway exists (reported in 28% of cases), it mainly involves joint consultations between pediatric and adult neurologists, followed by the transfer of medical records and patient referrals to the new center. In other cases, the transition simply consists of the pediatric center scheduling an appointment with the new specialist. The entire transition process takes a mean of 12 months, and in 30% of cases, caregivers report that it was not formally organized by the reference center. Additionally, the involvement of general practitioners in the transition process is generally not foreseen.

In adulthood, many treatments essential for rehabilitation and quality of life are discontinued: half of the caregivers surveyed report that their loved one is no longer receiving treatment or therapy, while smaller proportions continue to receive support from behavioral educators, nutritionists, physiatrists, cardiologists, or physical therapists.

Looking at the transition process, a particularly alarming finding concerns the emotional state most commonly reported by caregivers: fear, specifically related to concerns about inadequate care and dealing with their loved one's cognitive delays. Overall, caregivers perceive a decline in the quality of care when transitioning from pediatric to adult care, with the most critical issues in the patient journey being seizure management and daily condition management, exacerbated by a severe lack of communication and support. From an *administrative-bureaucratic dimension*, the transition often coincided with discontinuities in exemption renewals and care plans, representing a critical barrier to continuity of care.The difference in dropout rates between LGS patients (5/12; 40%) and DS patients (7/18; 38%) was not statistically significant [*χ*^2^(1) = 0.12, *p* = 0.73; 95% CI for difference: −12.5% to +16.3%].

To address these concerns, caregivers believe that the most urgent actions should focus on providing greater material and psychological support to families, as well as raising awareness among health care providers about the importance of listening to and respecting caregivers and patients.

### 3.4 Comparison of DEEs/LGS vs. DS organizational and management models

This section focuses on direct comparisons between DS and DEEs/LGS caregivers, while physicians' responses are reported only when relevant differences between syndromes were observed.

A comparative analysis of management models in the therapeutic areas of DEEs/LGS and DS revealed notable differences in how centers operate.

Regarding the transition to adulthood, the DS model appears to be more structured, with only 17% of DS centers lacking a formal transition pathway compared to 54% in the DEEs/LGS area. However, previous studies (7) have shown that many so-called structured pathways exist primarily on paper and fail to meet the actual care needs of patients and families during this critical phase. Moreover, transition pathways, where available, have existed in both areas for approximately 10 years, and in the DS model, they involve greater participation from general practitioners.

In terms of patient care and support services, DS patients have better access to management tools: 83% of DS centers offer remote telemonitoring for patients who cannot easily visit the center, and 66% provide psychological support services (compared to 43% for DEEs/LGS). However, the situation is reversed when it comes to training programs for condition and therapy management: only 17% of DS centers offer such training, compared to 69% of DEEs/LGS centers.

In terms of therapeutic choices, physicians report that the hospital drug formulary imposes greater restrictions on DS patients than on those with LGS and other DEEs. On a scale from 1 to 7, agreement with the statement “*At my center, the hospital drug formulary imposes limitations on therapeutic choices for LGS patients”* scores 3.15 for DEEs vs. 3.58 for DS. Probably, this is not only due to the formulary but also to the contraindications of a large group of drugs (sodium channel blockers) in Dravet syndrome due to its genetic defect. Cost factors, however, seem to pose a smaller barrier for DS patients, with agreement on the statement “*At my center, the cost of drugs limits therapeutic choices for LGS”* rated 3.23 for DEEs/LGS and 2.83 for DS. Although the mean values indicate a trend toward greater formulary restrictions for DS patients (mean 3.58) compared to DEEs/LGS (mean 3.15), this difference did not reach statistical significance [*t*(45) = 0.74, *p* = 0.46]. Similarly, the difference in perceived cost-related limitations (DEEs/LGS: 3.23 ± 1.4 vs. DS: 2.83 ± 1.3) was not statistically significant [*t*(45) = 1.02, *p* = 0.31]. Nevertheless, despite the absence of statistical significance, these directional trends remain noteworthy from an interpretative and organizational perspective. The slightly higher formulary restriction scores reported by physicians managing DS patients may reflect the presence of more institutionalized and protocol-driven care pathways, which, while ensuring treatment standardization, can also limit individual clinical discretion in therapeutic choices. Conversely, cost-related constraints appear somewhat less influential in DS management, possibly due to the consolidation of dedicated care networks and funding mechanisms that support long-term management. Although these findings should be interpreted descriptively, they reveal structural and behavioral dynamics that warrant further investigation beyond purely statistical significance.

**Figure 3:**
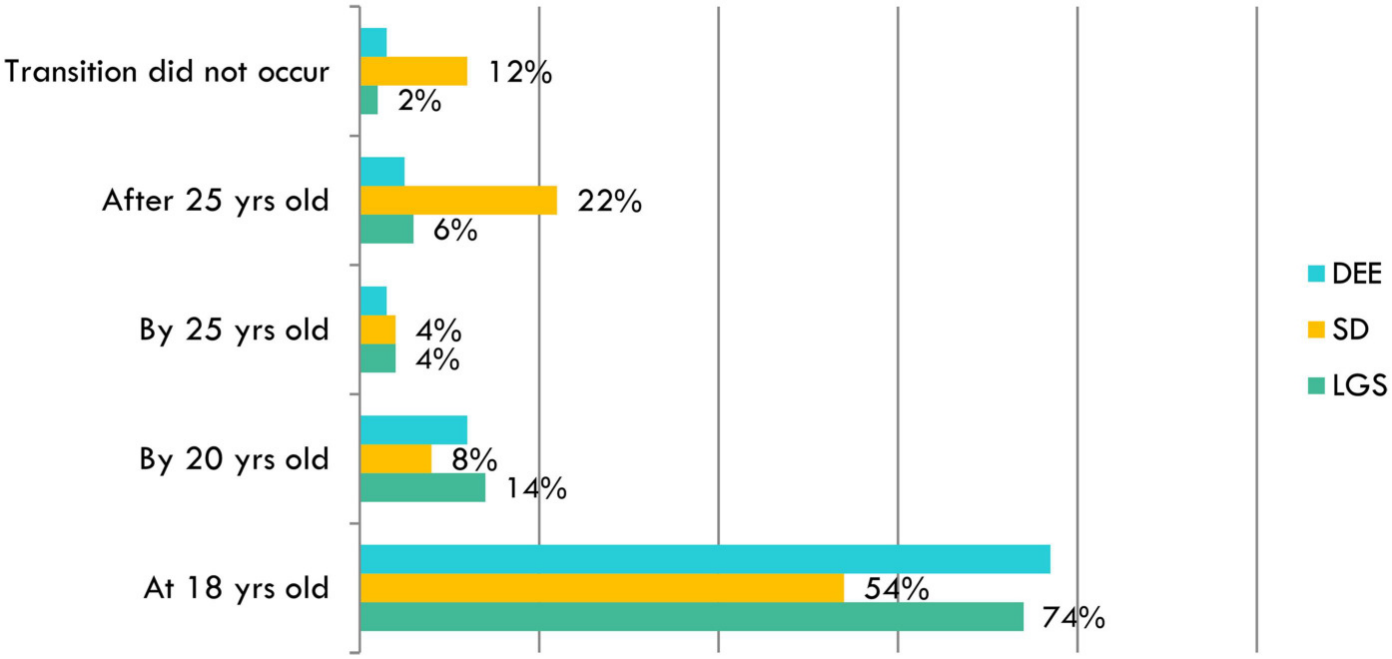
Comparison of the age at which the transition to an adult care center begins for different patient groups (DEEs/LGS and DS), based on physician reports: transition to adult care is delayed in DS patients, with only 54% transitioning at 18 years old, compared to 74% of LGS patients.

Regarding the transition age, the delayed shift to adult care in DS patients is confirmed (Figure 3). This difference can also be interpreted through a *political-institutional dimension*, as DS benefits from stronger advocacy, institutional recognition, and dedicated policies, which may partly explain the more structured but delayed transition models.

The proportion of patients transitioning after 25 years was significantly higher among DS patients (4/18; 22%) compared to DEEs/LGS patients (1/12; 6%) [*χ*^2^(1) = 6.85, *p* = 0.009; Cramer's *V* = 0.28], suggesting a moderate association between syndrome type and delayed transition.

While 54% of DS patients (10/18) transitioned at 18 years of age, a significant proportion (4/18; 22%) transitioned after 25 years, compared to only 5%–6% in DEEs/LGS. Additionally, 12% of (2/18) DS cases had no documented transition at all.

Finally, the *cultural dimension* emerged in the way families interpreted and negotiated illness trajectories: while DS was more frequently framed within a recognized and institutionalized condition, DEEs/LGS remained less visible, with families often perceiving marginalization and lower societal awareness.

In terms of perceived attitude of families toward transition to adult care centers, DS families appear to be significantly more accepting of the process (60% of cases vs. 14% of DEEs/LGS patients). For both groups, initial resistance is primarily due to fear of losing their trusted specialist. However, for LGS and DEEs patients, this concern is compounded by the lack of an integrated transition pathway to adult care. In the case of DEEs/LGS, 14% of patients strongly resist the transition, and 11% do not accept it, while the majority of families are initially hesitant (61%).

The dropout rate from adult care centers is similar across conditions, with clinicians estimating that approximately 40% of patients discontinue care within the first 18 months of transition. After this period, patient management is primarily home-based, often under the supervision of a private specialist.

Regarding the main challenges encountered in patient care, physicians report that diagnostic difficulties are a major issue for both DEEs/LGS and DS. However, from the families' perspective, physicians identify seizure control as the primary difficulty for DEEs/LGS patients, who continue to experience frequent seizures into adulthood, whereas communication and psycho-cognitive issues pose greater challenges for DS patients.

### 3.5 The patient journey: an integrated perspective from physicians and caregivers

The study also aimed to reconstruct key stages of the patient journey for Dravet Syndrome (DS), as reported by pediatric neuropsychiatrists and adult neurologists, to compare it with findings from previous research (9) and with the care pathway for DEEs/LGS patients. For the latter, a unified journey was mapped, highlighting specific differences in the case of LGS patients.

Clinicians report an mean diagnostic timeframe of 4 months from the onset of first symptoms (8 months for DEEs and 7 months for LGS according to neurologists, and 13 months for DEEs and 16 months for LGS according to pediatric neuropsychiatrists). However, they rarely indicate that this timeframe exceeds 12 months, contrasting with caregiver reports and previous case studies, which suggest significant diagnostic delays spanning several years. In the case of DEEs and LGS, a larger proportion of clinicians acknowledge diagnostic delays beyond 12 months: 19% of DEEs patients and 24% of LGS patients according to neurologists, and 29% of DEEs patients and 22% of LGS patients according to pediatric neuropsychiatrists.

For DS, diagnostic delays are reported as occasional in 67% of cases, compared to 73% for DEEs/LGS according to neurologists and 56% according to pediatric neuropsychiatrists. The consequences of these delays include a negative impact on development and seizure control, non-specific or only partially effective therapeutic choices, and delays in receiving genetic test results. These findings align with the different onset patterns of the two syndromes: DS symptoms appear early and present distinct clinical features, whereas LGS takes longer to fully manifest, delaying the confirmation of diagnostic criteria.

The most commonly performed diagnostic tests include sleep and awake EEG (for almost all DEEs and LGS patients), awake EEG, MRI scans. Also, genetic testing is often performed. These procedures primarily involve pediatric neuropsychiatrists, neurologists, psychologists, and primary care pediatricians (Table 1 and 2). The diagnosis is made by the pediatric neuropsychiatrist, but a psychologist is present in only 25% of cases.

**Table 1 and 2:** Comparison of diagnostic tests performed on suspected DEEs/LGS patients (right) vs. DS patients (left), based on physician reports.

Tests for patients with suspected DS	%	Tests for patients with suspected DEEs/LGS	LGS	DEEs
EEG (awake)	43%	EEG (awake)	59%	57%
EEG (awake and sleep)	75%	EEG (awake and sleep)	95%	97%
Brain MRI	87%	Brain MRI	97%	98%
Physical examination	68%	Physical examination	22%	24%
Laboratory tests	50%	Laboratory tests	84%	81%
Magnetic Resonance Imaging (MRI)	87%	Magnetic Resonance Imaging (MRI)	51%	49%
Genetic panels	37%	CT scan	40%	38%
Array CGH	12%	Genetic test	24%	22%
CT scan	31%			
Genetic tests (SCN1A and gene panel tests)	43%			
Other	2%			

Routine follow-up visits occur at a mean interval of 4 months for DS patients and are conducted almost exclusively in person at the healthcare facility. Remote consultations are rare, with 7% occurring via telephone and 4% via a dedicated telehealth platform.

For LGS patients, the mean follow-up frequency is 8 months, while for DEEs patients it is 9 months, with no use of dedicated telehealth platforms reported.

**Figure 4:**
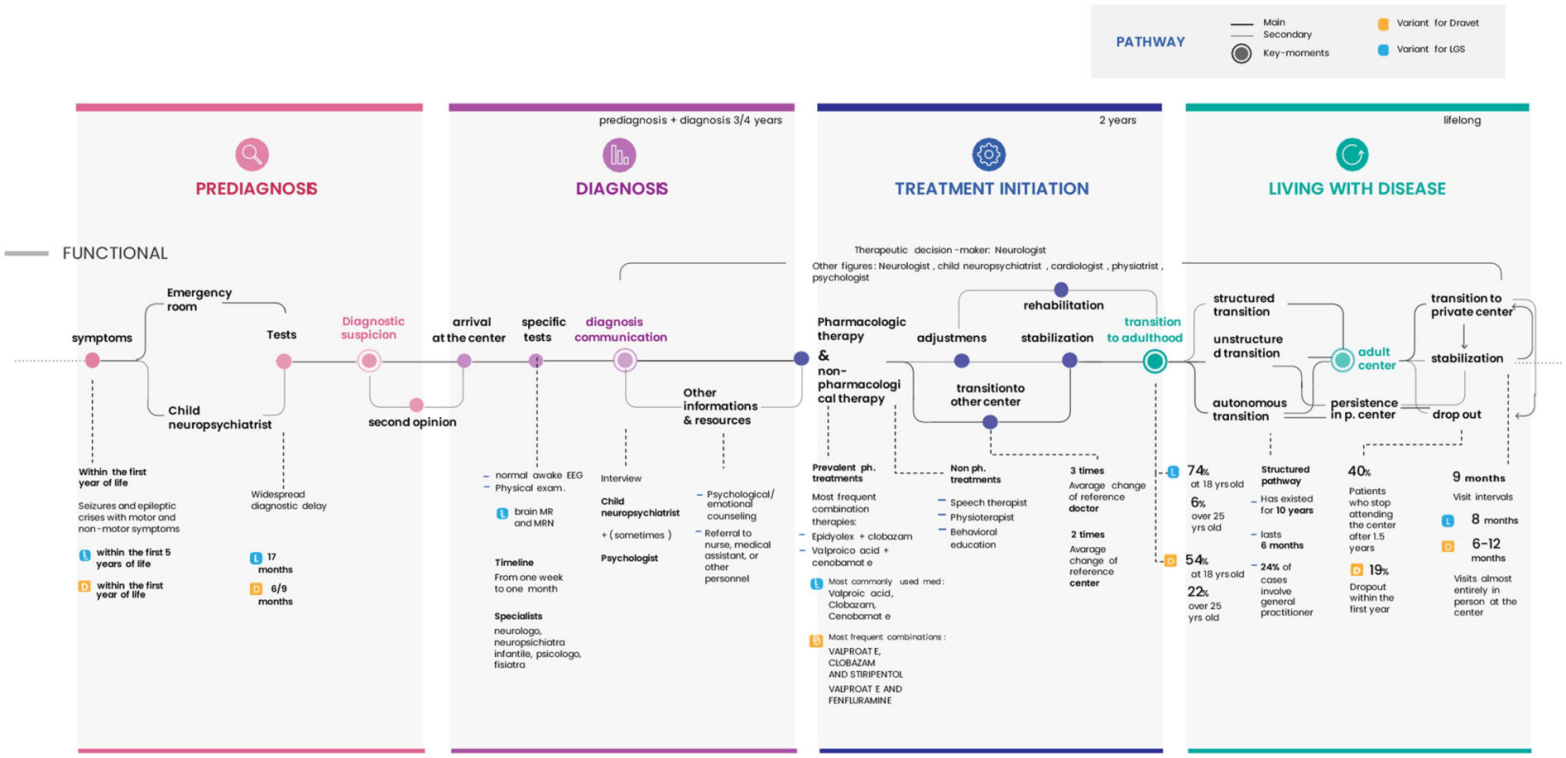
Comparative patient journey for DS and DEEs/LGS structured into four key phases: pre-diagnosis, diagnosis, treatment initiation, and long-term management.

The collected data allowed for the reconstruction of a comparative Patient Journey between DS and DEEs/LGS patients (Figure 4), structured into four key phases: pre-diagnosis, diagnosis, treatment initiation, and long-term management. This visual representation provides a concise overview of the complex steps patients and their families must navigate.

Based on physician and caregiver reports, the main differences between the two patient journeys include:

1. Pre-diagnosis:
  - Later symptom onset for DEEs/LGS (DS: within the first year of life; DEEs/LGS: within the first few years of life).
  - Longer diagnostic delays for DEEs/LGS (DS: diagnosis within 6–9 months; DEEs/LGS: diagnosis between 13 and 17 months).
2. Diagnosis:
  - Greater reliance on MRI scans for LGS patients.
3. Treatment Initiation:
  - Different therapeutic approaches.
4. Long-Term Management:
  - Later transition to adult specialists for DS (DEEs/LGS: transition at 18 years in 74%–77% of cases, after 25 years in 5%–6%; DS: transition at 18 years in 54% of cases, after 25 years in 22%).
  - High dropout rates from adult care centers for all conditions (DS: 19% dropout rate within the first year; DEEs/LGS: 27% dropout rate).
  - Comparable follow-ups frequency across conditions (mean of 9 months).

Based on caregiver survey data, several key aspects of the DEEs/LGS patient journey have emerged. Notably, DEEs/LGS patients first exhibited symptoms at a mean age of 2 years, compared to within the first year of life for DS patients. Following the onset of symptoms, 47% of families sought emergency care as their first point of contact. Before reaching a specialist, families reported seeing a mean of three different health professionals. Additionally, caregivers indicate that the mean time from symptom onset to diagnosis is approximately two years, which contrasts with specialist-reported data. This discrepancy suggests that clinicians may underestimate the diagnostic delay for this condition. The same trend is observed in DS patients, where diagnostic delays appear substantial from caregivers' perspectives (including cases of diagnosis in adolescence (9), while physicians report a mean delay of only four months. The consequences of this delay, as perceived by caregivers, are mainly stress and frustration, but also in the need to travel far from home and the delay in starting the right therapy.

**Figure 5:**
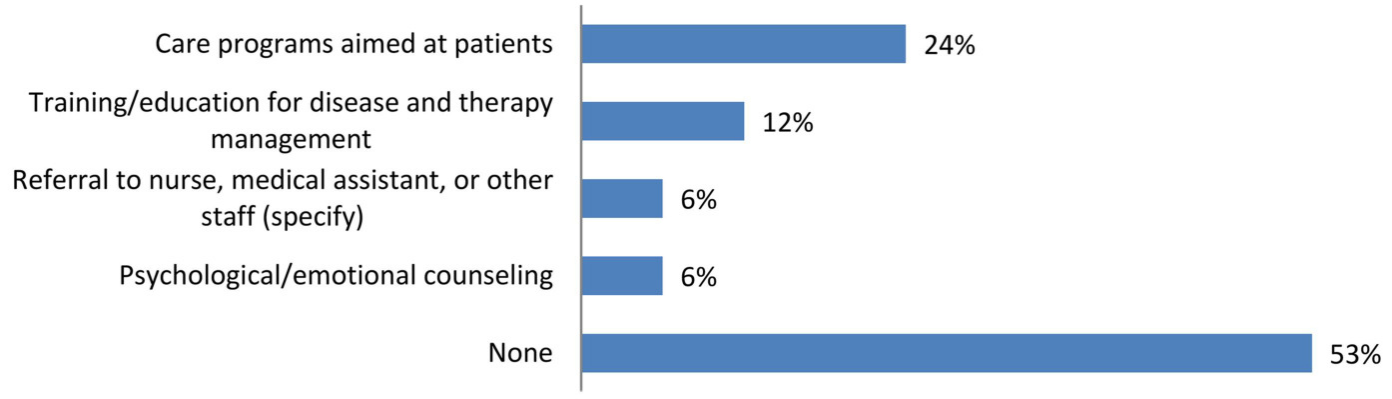
Information and resources provided to families following diagnosis: the majority of caregivers for DEEs/LGS patients reported they did not receive any services after the diagnosis.

The survey confirms what emerged regarding the communication of the diagnosis, which was made by the child neuropsychiatrist in the absence of the psychologist in 75% of the cases. However, the perspective differs regarding the services provided post-diagnosis, where the majority of caregivers (53%) state that, in the case of DEEs/LGS, they did not receive any type of service, while a significant portion (24%) received care programs aimed at patients (Figure 5).

Very limited is also reported home care, where in 70% of cases the structures did not implement any resources or support services for this type of management, while a smaller proportion reported having access to rehabilitation services. On the other hand, communication regarding compensations and exemptions to which the patient could access was timely, and this was also the case for patients with DS. Up until the time of the interview, most caregivers stated that they had changed their referring doctor a mean of 3 times, mainly due to organizational changes within the facility, but also out of the desire to be properly followed and to explore new paths and therapeutic approaches; in total, the reference center changed for 53% of patients with a mean of 2.5 facilities, mainly to obtain a second opinion or for reasons similar to those mentioned for changing the specialist. The patients of the interviewed caregivers are currently followed mainly by individual specialists, in 53% of cases by a neurologist, in 41% of cases by a child neuropsychiatrist, and a small proportion by a general practitioner (6%). Multidisciplinary teams are reported only in 12% of cases.

## 4 Discussion

Comparative analysis of DEEs/LGS and DS management models reveals significant differences in patient care pathways and treatment approaches, highlighting critical gaps that warrant closer examination. In addition to descriptive comparisons, statistical analyses confirmed that some differences were significant. For example, caregiver training was markedly more frequent in DEEs/LGS centers, while delayed transitions beyond age 25 were significantly more common in DS patients. Conversely, dropout rates did not differ significantly across conditions. These findings strengthen the robustness of the results and highlight areas where organizational disparities are statistically substantiated.

DS care centers are better equipped with services such as telemonitoring and psychological support, whereas DEEs/LGS centers prioritize caregiver training for disease and therapy management, with a broader range of educational programs available. This contrast reflects a fundamental difference in care priorities: DS centers tend to adopt a more protective, hospital-centered approach, whereas DEEs/LGS care emphasizes caregiver empowerment and home-based management. This distinction is also evident in the prolonged retention of DS patients in pediatric care centers, with a higher proportion of DS patients remaining in pediatric facilities beyond the age of 25.

Another key area of comparison is therapeutic decision-making. Restrictions imposed by the hospital drug formulary appear to impact DS patients more than DEEs/LGS patients. At the same time, the cost of medications poses a greater barrier for DEEs/LGS care, underscoring the need for more flexible, patient-centered policies for both conditions.

The transition from pediatric to adult care is a critical phase for DEEs, LGS, and DS patients, presenting substantial challenges for both patients and caregivers (11, 12). The study highlights significant variability in transition models, with a lack of standardization that fails to meet the specific needs of these patients. Moreover, regarding family attitudes toward transition to adult centers, clinicians observe that families of patients with DEEs/LGS tend to be less accepting of transition compared to patients with DS, an attitude that may be related to the lack of structured pathways for these therapeutic areas. The initial resistance, common to both patient categories, is often due to the fear of losing the trusted specialist, but in the cases of patients with LGS and DEEs, it is exacerbated precisely by the absence of an integrated pathway for adult care. Additionally, the dropout rate from adult centers is very high for all patient categories, after which the patient is primarily managed at home with the support of a private specialist, resulting in most cases in a lack of the integrated and multidisciplinary management that these patients particularly need.

The urgency and dramatic nature of the action required by this data is clear: diagnostic delays, the lack of a structured transition pathway, and the abandonment of adult centers can have a significantly negative impact on the entire clinical care pathway. In particular, it can lead to prolonged clinical and therapeutic instability, negatively influencing, in pediatric age, the planning of the transition, and in adulthood, the optimal long-term management of the patient (19).

Furthermore, findings on the quality of life of patients and families with DEEs, LGS, and DS reveal how the quality of life of the families of patients with such conditions is significantly affected by the illness, with a high social burden and a severe negative impact on the emotional health of caregivers and siblings: many families indeed face social isolation, job loss, and, in some cases, separation or divorce due to the challenges associated with caregiving (8).

Research demonstrates that caregivers experience significantly elevated levels of stress, anxiety, and depression, with studies reporting history of clinical depression treatment and post-traumatic stress disorder in DS and LGS caregivers (20, 21). The physical burden is equally substantial, as constant vigilance required for managing uncontrolled seizures and developmental disabilities, combined with sleep deprivation due to nocturnal seizures, leads to chronic exhaustion and physical strain, particularly affecting caregivers' back and shoulder health when assisting non-ambulatory patients (9–22). Also, social functioning is severely compromised, with studies showing that most of DS caregivers score below national averages in social domains, reflecting interpersonal relationship difficulties and social stigma associated with caring for a child with a rare epileptic disorder (21, 22).

Therefore, it is essential to take measures aimed at affirming the crucial role of caregivers, including siblings, by raising awareness among institutions and society at large, in order to provide them with adequate recognition and psychological and social support to cope with the difficulties associated with these conditions.

The implementation of structured and integrated models, patient services, and well-defined and tested transition pathways in all centers can indeed be initiated starting, for example, from best practices implemented in other centers of excellence, and whose models can be disseminated and adapted to different local realities. In light of these findings, several recommendations can be made to facilitate the development of structured transition models. First, centers should adopt shared protocols that clearly define the timing and steps of transition, ensuring early discussion with families and progressive involvement of adult specialists. Establishing a dedicated transition coordinator represents a critical organizational change that should initiate the process at least two years before the patient reaches 18 years of age, drawing from the more structured DS model. This early initiation allows sufficient time to address the complex medical and social needs of these patients while building trust with adult care providers.

Second, the establishment of formal multidisciplinary teams—including neurologists, pediatric neuropsychiatrists, psychologists, and rehabilitation professionals—is essential to guarantee continuity of care across life stages. The implementation of mandatory joint consultations between pediatric and adult neurologists constitutes another essential component, ensuring therapeutic continuity and potentially reducing the concerning 40% dropout rate observed within the first months of transition. These collaborative meetings should focus not only on medical handover but also on addressing family concerns about care quality decline, which represents the primary source of anxiety during this critical phase. Drawing on the experience of DS centers, the implementation of telemonitoring services and psychological support for both patients and caregivers should be extended to DEE/LGS pathways, where such services are often lacking. Comprehensive family training programs addressing adult disease management should be expanded beyond their current availability in DEE/LGS centers to include psychological support services, learning from the higher provision rates demonstrated in DS centers.

Additionally, caregivers should be formally recognized as integral members of the care team through structured involvement in clinical decision-making processes and transition planning committees, including participation in multidisciplinary meetings and formalized communication protocols between caregivers and healthcare providers. Support for caregivers through educational programs, psychological support programs, and support groups can improve their understanding and management of the condition while alleviating their social and emotional burden (23, 24), thus helping to preserve the social network of the patient and family.

While these considerations highlight important directions for improving care and transition management, it is also necessary to acknowledge some limitations of the present study, which should be taken into account when interpreting the findings. First, despite the rarity of these conditions, the sample of physicians and caregivers may not be representative at the national level. Moreover, as the data are self-reported, responses from both caregivers and physicians may be subject to recall or social desirability bias, which could have influenced the results. Finally, the study shows a concentration of participating centers in Northern and Central Italy, and this geographic imbalance may limit the generalizability of the findings to the entire national context. Additionally, as this research was conducted within the Italian healthcare system, the applicability of these findings to broader European contexts may be limited. Addressing these limitations in future studies will be crucial to consolidate the evidence base and to design effective, equitable, and sustainable care models capable of improving both clinical outcomes and the quality of life of patients and their families.

## Data Availability

The raw data supporting the conclusions of this article will be made available by the authors, without undue reservation.
