# Supplementary material for: Organizational and behavioral models in the management of patients with developmental and epileptic encephalopathy, Lennox-Gastaut syndrome and Dravet syndrome in Italy: a focus on the transition from pediatric to adult care
Source: Front Health Serv. 2025 Nov 7;5:1632564. doi: 10.3389/frhs.2025.1632564 (PMC12634588; doi:10.3389/frhs.2025.1632564)
Supplement: Supplementary file 1 [file Supplementaryfile1.docx]

**Fig.1**


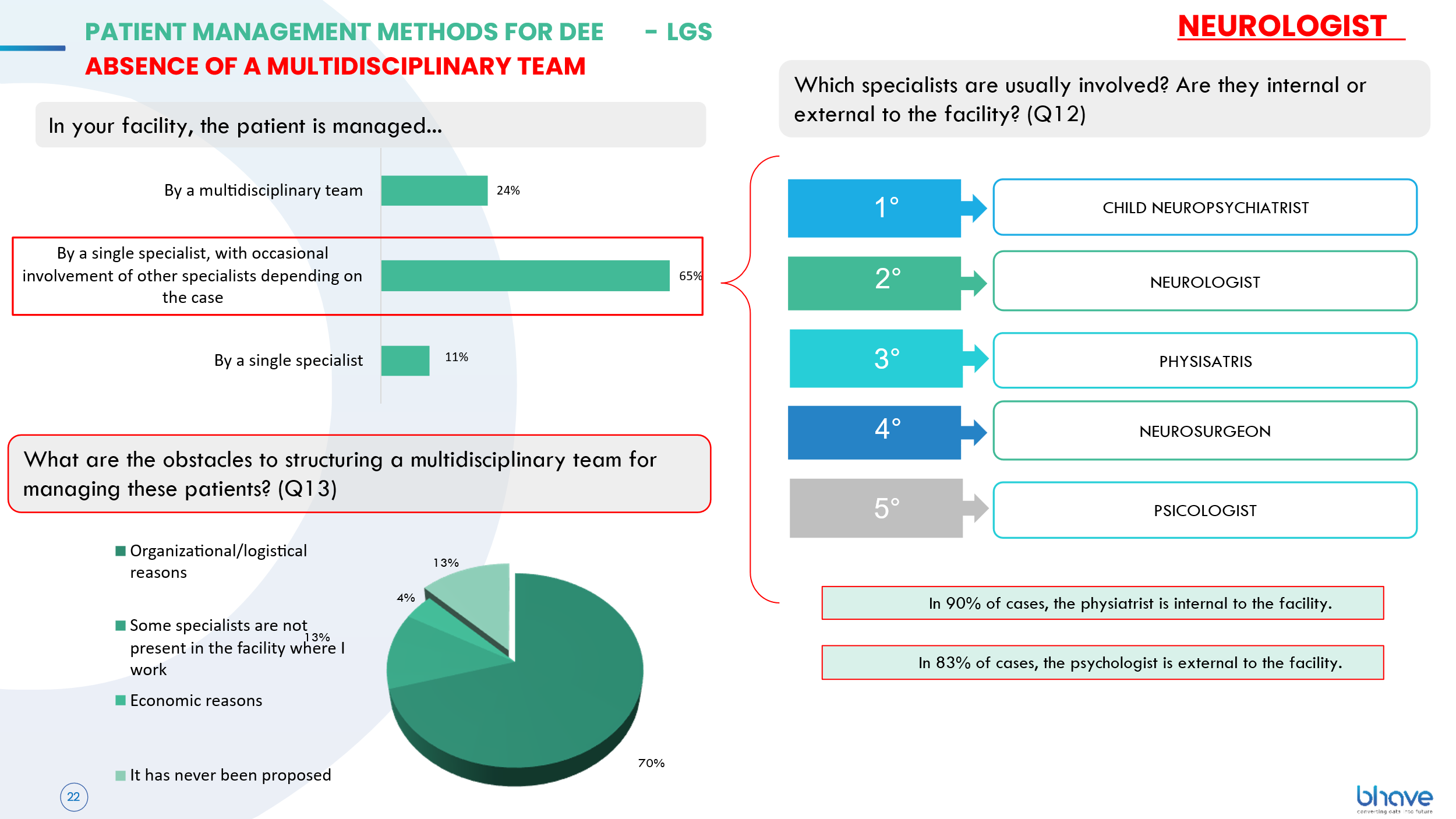


*Fig 1. Management of DEEs and LGS patients in the absence of a multidisciplinary team and specialists involved in their care.*

Fig.2

*Fig 2. Management of the transition to adult care based on reports from patients currently undergoing the transition or receiving care at an adult center*

*Fig. 3*

*Fig 3. Comparison of the age at which the transition to an adult care center begins for DS patients (left) and DEE/LGS patients (right), based on physician reports.*

*Fig. 4.*


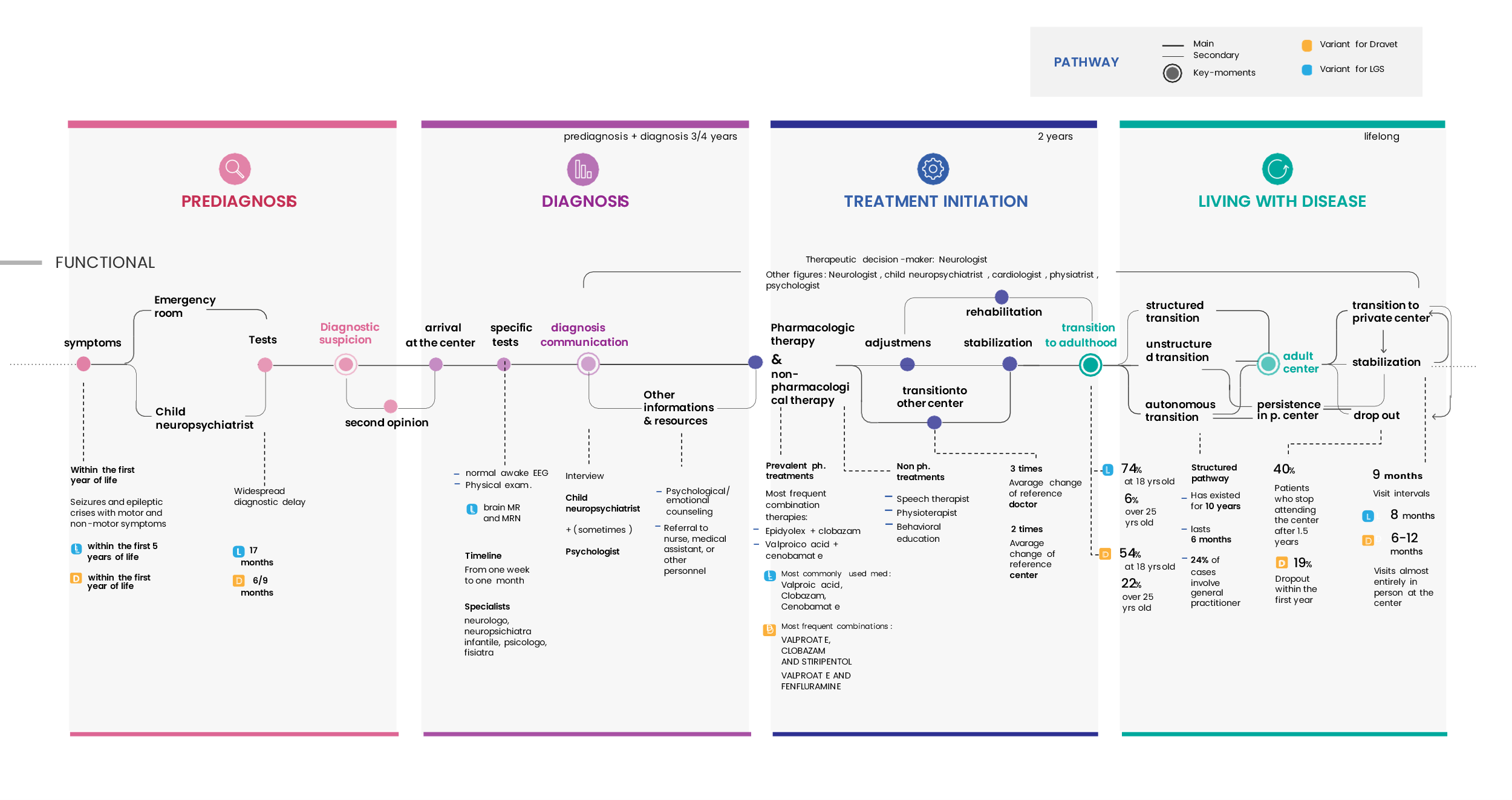


*Fig 4: Comparative Patient Journey for DS and DEEs/LGS*

*Fig. 5*

*Fig 5. Information and resources provided to families following the diagnosis formulation.*

*Table 1 and Table 2*

| Tests for patients with suspected DS | **Distribuzione %** |
| --- | --- |
| EEG (awake) | 43% |
| EEG (awake and sleep) | 75% |
| Brain MRI | 87% |
| Physical examination | 68% |
| Laboratory tests | 50% |
| Magnetic Resonance Imaging (MRI) | 87% |
| Genetic panels | 37% |
| Array CGH | 12% |
| CT scan | 31% |
| Genetic tests (SCN1A and gene panel tests) | 43% |
| Other | 2% |

| Tests for patients with suspected DEE/LGS | **LGS** | **DEE** |
| --- | --- | --- |
| EEG (awake) | 59% | 57% |
| EEG (awake and sleep) | 95% | 97% |
| Brain MRI | 97% | 98% |
| Physical examination | 22% | 24% |
| Laboratory tests | 84% | 81% |
| Magnetic Resonance Imaging (MRI) | 51% | 49% |
| CT scan | 40% | 38% |
| Genetic test | 24% | 22% |

*Table 1 and 2. Comparison of diagnostic tests performed on suspected DEE/LGS patients (right) vs. DS patients (left), based on physician reports.*
