# Supplementary material for: Organizational and behavioral models in the management of patients with developmental and epileptic encephalopathy, Lennox-Gastaut syndrome and Dravet syndrome in Italy: a focus on the transition from pediatric to adult care
Source: Front Health Serv. 2025 Nov 7;5:1632564. doi: 10.3389/frhs.2025.1632564 (PMC12634588; doi:10.3389/frhs.2025.1632564)
Supplement: Supplementary file 2 [file Datasheet1.docx]

**Questionario**

**ZONA GEOGRAFICA** NORD OVEST |_1_| (25% Interviste Target)

NORD EST |_2_| (25% interviste per Target)

CENTRO |_3_| (25% interviste per Target)

SUD E ISOLE |_4_| (25% Interviste per Target)

1. Lei è un…

❑1 Caregiver di soggetto con Sindrome di Lennox-Gastaut (LGS) o con Encefalopatia Epilettica e dello Sviluppo (DEE) 🡪 proseguire

❑2 Conoscente/amico di un paziente con LGS/DEE 🡪 (ringraziare e chiudere l’intervista)

❑3 Caregiver di soggetto con epilessia 🡪 (ringraziare e chiudere l’intervista)

❑4 Altro 🡪 (ringraziare e chiudere l’intervista)

1. Quale è il rapporto di parentela che la lega al soggetto con SD?

❑1 Genitore 🡪 proseguire

❑2 Fratello/Sorella 🡪 proseguire

❑3 Altro (specificare):______ 🡪 proseguire

❑4 Nessun rapporto di parentela 🡪 proseguire

1. Se (Q3=4) Da quanti anni assiste un soggetto Dravet? |__|__| anni (Se < 1 🡪 ringraziare e chiudere)
2. Quanti anni ha il suo assistito?

| 1. 0-14 anni | 🡪 proseguire per N=2 |
| --- | --- |
| 1. 15-17 anni | 🡪 proseguire per N=2 |
| 1. 18-29 anni | 🡪 proseguire per N=4 |
| 1. Più di 30 | 🡪 proseguire per N=2 |

1. Il suo assistito viene attualmente seguito* da:

❑1 Centro pediatrico – neuropsichiatra pediatrico 🡪 proseguire

❑2 Centro dell’adulto – neurologo 🡪 proseguire

❑3 Altro (specificare):______ 🡪 proseguire

***Con “seguito” si intende lo svolgimento di almeno una visita/anno

**FASE 1 – PATIENT JOURNEY E MODELLI DI ASSISTENZA**

***Finalità:***  *Tracciare il percorso dal momento in cui si sono presentati i primi sintomi, identificando gli snodi e i momenti significativi*

1. A quanti anni/mesi di età sono apparsi i primi sintomi della patologia nel suo assistito?

I primi sintomi si sono manifestati a **Q15a1**|__|__|__| **Q15a2**❑ mesi ❑anni

1. A quale figura/struttura vi siete rivolti per primo una volta che si sono manifestati questi sintomi?

❑ Pediatra di base

❑ Pronto Soccorso

❑ Specialista pediatra

❑ Neuropsichiatra infantile/ Neurologo pediatra

❑ Guardia medica

❑ Non so/ Non ricordo

❑ Altro (specificare):______________

1. Con quante figure mediche si è interfacciato il suo assistito prima di arrivare allo specialista giusto?

Prima di arrivare allo specialista giusto, il mio assistito si è interfacciato con |__|__|__| figure mediche

1. Dopo quanto tempo dalla comparsa dei primi sintomi le è stata data diagnosi di LGS/DEE?
   Solitamente la diagnosi arriva dopo **Q18a2**|__|__|__| **Q18a2** ❑ mesi ❑anni dalla comparsa dei primi sintomi
2. Se c’è stato un ritardo nella diagnosi, che conseguenze ha avuto? (Risposta multipla)
3. Ritardo nell’intraprendere la terapia corretta
4. Aggravarsi della patologia
5. Stress e frustrazione per i familiari
6. Viaggio fuori dal proprio territorio di residenza per effettuare altre visite
7. Dispendio economico nell’effettuare più esami/visite
8. Disagi per i caregiver nella gestione del lavoro
9. Non c’è stato ritardo nella diagnosi
10. Altro (specificare):_______________
11. Quale figura ha comunicato la diagnosi di LGS/DEE?

❑ Neurologo

❑ Neuropsichiatra

❑ Fisiatra

❑ Neurofisiatra

❑ Non so/non ricordo

❑ Altro (specificare):____________

1. wEra presente lo psicologo durante la comunicazione della diagnosi? ❑ Sì ❑ No ❑ Non so/ non ricordo
2. Quali informazioni o risorse vi sono state fornite dopo che la diagnosi è stata formulata?

| **RISORSE** |  |
| --- | --- |
| 1. Programmi di supporto ai pazienti (PSP) | ❑ |
| 1. Training/educazione per la gestione della patologia e della terapia | ❑ |
| 1. Contatti con organizzazioni dei pazienti (OP) | ❑ |
| 1. Opuscoli educativi | ❑ |
| 1. Rinvio a infermiere, assistente medico o altro personale (specificare) | ❑ |
| 1. Consulenze psicologiche/ emotive | ❑ |
| 1. Altro (Specificare):_____________ | ❑ |

1. Siete stati informati da subito delle **esenzioni/indennizzi** a cui il suo assistito ha diritto?

❑ Sì, da subito

❑ Non subito, è stato necessario chiederlo

❑ No, è stata l’Associazione pazienti a fornirci queste informazioni

❑ Non so/ non ricordo

1. Quanti **medici di riferimento** ha cambiato il suo assistito dalla diagnosi ad oggi?
2. È rimasto sempre lo stesso
3. È stato cambiato |__|__| volta/ volte

**Q24a1:** (Se Q24=2) Quali sono stati i motivi di questo cambiamento?

____________________________________________________________

1. Quanti **Centri di riferimento** ha cambiato il suo assistito dalla diagnosi ad oggi?
2. È rimasto sempre lo stesso
3. È stato cambiato |__|__| volta/ volte
4. Attualmente non frequenta più il centro di riferimento

**Q25a1:** (Se Q25=2) Quali sono stati i motivi di questo cambiamento?

____________________________________________________________

1. Attualmente chi è il medico di riferimento del suo assistito?
2. Neurologo ❑ del centro di riferimento ❑ privato
3. Neuropsichiatra ❑ del centro di riferimento ❑ privato
4. Pediatra/ medico di base
5. Altro (specificare):____________
6. Attualmente il suo assisto viene seguito…
7. Da un singolo specialista
8. Da un’equipe multidisciplinare interna al centro di riferimento
9. Dallo specialista di riferimento + altre figure sul territorio che si occupano di altri trattamenti (es. fisiatra, fisioterapista, logopedista, ecc)
10. Altro (specificare):_________________________________________________
11. Considerando le seguenti **terapie farmacologiche**, quali sono state seguite dal suo assistito? Quale è stata la prima terapia e qual è la terapia attuale che segue? (Risposta multipla)

| **TERAPIE FARMACOLOGICHE** | **PRIMA TERAPIA** | **TERAPIA ATTUALE** | |  |
| --- | --- | --- | --- | --- |
| ❑Felbamato (es. Taloxa) | ❑ | ❑ | |  |
| ❑Lamotrigina (es. Lamictal) | ❑ | ❑ | |  |
| ❑Topiramato (es. Topamax, Epitomax, Topiramat-Cilag, ecc) | ❑ | ❑ | |  |
| ❑Levetiracetam (es. Keppra) | ❑ | ❑ | |  |
| ❑Rufinamide (es. Inovelon) | ❑ | ❑ | |  |
| ❑ Acido valproico (es. Depakin) | ❑ | ❑ | |  |
| ❑ Fenfluramina (es. Fintepla) | ❑ | ❑ | |  |
| ❑ Fenobarbitale (es. Gardenale, Luminale) | ❑ | ❑ | |  |
| ❑ Progabide | ❑ | ❑ | |  |
| ❑ Gabapentin (es. Keneil, Neurontin, ecc) | ❑ | ❑ | |  |
| ❑ Clobazam (es. Frisium, Epaclob) | ❑ | | ❑ | |
| ❑ Carbamazepina (es. Tegretol) | ❑ | | ❑ | |
| ❑ Fenitoina (es. Dintoina) | ❑ | | ❑ | |
| ❑ Cannabidiolo | ❑ | | ❑ | |
| ❑ Altro (Specificare):_____________ | ❑ | | ❑ | |

1. Quali altri **trattamenti e terapie** vengono seguiti dal suo assistito? (Risposta multipla)
2. ❑Logopedista
3. ❑Psicologo
4. ❑Fisioterapista
5. ❑Cardiologo
6. ❑Fisiatra
7. ❑Nutrizionista
8. ❑Educatore comportamentale
9. ❑Altro (specificare):____________
10. Con che frequenza in numero di mesi avvengono in media le **visite di controllo** per il suo assistito?

In media ogni |__|__| mesi.

1. Quali sono le modalità con cui avvengono le visite di controllo? Indicare una stima della percentuale con cui si verificano le seguenti modalità. La somma delle % deve fare 100

|__|__|__| % in presenza nel centro di riferimento

|__|__|__| % in presenza in un'altra struttura (specificare________)

|__|__|__| % in presenza a domicilio

|__|__|__| % online con piattaforma di telemedicina dedicata(specificare___)

|__|__|__| % in videochiamata (es. WhatsApp, Zoom…)

|__|__|__| % telefonicamente

|__|__|__| % Altro specificare ______________

1. Il centro di riferimento da cui il suo assistito è seguito attualmente, ha messo in campo delle risorse o servizi che la aiutino nella gestione domiciliare della patologia?
2. No
3. Sì **Q27a1:** Quali? ❑Riabilitazione ❑ Terapia domiciliare ❑ Visite a distanza/Televisite ❑ Sistemi di monitoraggio a distanza ❑Altro (specificare):__________

(Se Q4= b, c, d)

**FASE 2 – TRANSIZIONE ALL’ETA’ ADULTA**

***Finalità:***  *Evidenziare i modelli assistenziali nella gestione del passaggio dall’età pediatrica all’età adulta*

1. Attualmente il suo assistito è in cura presso….
2. Un centro di riferimento pediatrico
3. Un centro di riferimento per l’adulto
4. Sta effettuando il passaggio dal centro pediatrico al centro dell’adulto
5. Altro (specificare):_________________

(Se Q28=1)

1. **qqQ29a.** Nel centro pediatrico in cui viene seguito il suo assistito, come viene gestito il momento di **passaggio all’età adulta**?
2. Al momento questo tema non viene affrontato **Q29a1.** Come mai?____________________________
3. Esiste un percorso di transizione dal centro pediatrico al centro dell’adulto di cui sono a conoscenza e che il mio assistito dovrà seguire
4. Non esiste un percorso di transizione codificato: il passaggio è in carico alle famiglie
5. Il mio assistito verrà seguito dal centro pediatrico finché non ci sarà la necessità di cambiare centro
6. Altro (specificare):_____________________

**Q29b.**  In base alle sue informazione, a che età avviene/avverrà il passaggio al centro dell’adulto per il suo assistito?

1. A partire dai 18 anni
2. Dopo i 20 anni
3. Dopo i 25 anni
4. Il passaggio si effettuerà solo in caso di bisogno
5. Quando io/ la famiglia del mio assistito troverà un centro adatto
6. Non so

(Se Q28=2 o 3)

1. Quale delle seguenti affermazioni **rispecchia meglio la sua esperienza** nel passaggio al centro dell’adulto?
2. Il passaggio al centro dell’adulto è stato avviato dal centro di riferimento pediatrico quando il mio assistito si avvicinava/ aveva raggiunto la maggiore età
3. Il passaggio al centro dell’adulto è stato posticipato dal centro pediatrico non per scelta del mio assistito
4. Il passaggio al centro dell’adulto è stato posticipato per scelta della mia famiglia/ della famiglia del mio assistito
5. Nel centro di riferimento pediatrico non esiste un percorso di transizione codificato: il passaggio è in carico alle famiglie che devono individuare il centro
6. Altro (specificare):___________________________________________
7. Se organizzato dal centro di riferimento, in quali step si articola il percorso di transizione verso il centro dell’adulto?
8. ❑Il percorso di transizione non è stato organizzato dal centro di riferimento
9. ❑ Si articola nei seguenti passaggi:
10. ____________________
11. ____________________
12. ____________________
13. ____________________
14. ____________________
15. ____________________
16. ____________________

**Q31a1.** È previsto il coinvolgimento del medico di base? ❑ Sì ❑No ❑ Non so

**Q31a2.** Quando dura complessivamente il periodo di passaggio? |__|__|__| ❑ giorni ❑ mesi

1. Quali **trattamenti e terapie**  avete proseguito/ verranno proseguiti in età adulta? (Risposta multipla)

❑Logopedista

❑Psicologo

❑Fisioterapista

❑Cardiologo

❑Fisiatra

❑Nutrizionista

❑Educatore comportamentale

❑Altro (specificare):____________

1. Come valuta complessivamente la **gestione e l'assistenza ricevuta nel passaggio** dal centro pediatrico al centro dell’adulto? (Esprimere una valutazione da 1 a 7)

| **1** | **2** | **3** | **4** | **5** | **6** | **7** |
| --- | --- | --- | --- | --- | --- | --- |
| ❑1 | ❑2 | ❑3 | ❑4 | ❑5 | ❑6 | ❑7 |

**FASE 3 – CONCLUSIONI**

1. Quali sono **le prime tre criticità che ha incontrato** nella gestione del suo assistito?

1° |_____________________________|

2° |_____________________________|

3° |_____________________________|

1. Quali sono **le tre azioni/ iniziative più urgenti** da mettere in campo per supportare i pazienti e i caregiver durante il percorso di cura?

1° |_____________________________|

2° |_____________________________|

3° |_____________________________|

1. Come valuta complessivamente la qualità dell’assistenza ricevuta durante il percorso di cura del suo assistito? (Esprimere una valutazione da 1 a 7)

| **1** | **2** | **3** | **4** | **5** | **6** | **7** |
| --- | --- | --- | --- | --- | --- | --- |
| ❑1 | ❑2 | ❑3 | ❑4 | ❑5 | ❑6 | ❑7 |

1. Come valuta **l’attenzione che il SSN pone sulle malattie rare** come l’Encefalopatia Epilettica e dello Sviluppo (DEE) o la Sindrome di Lennox-Gastaut (LGS)? (Esprimere una valutazione da 1 a 7)

| **1** | **2** | **3** | **4** | **5** | **6** | **7** |
| --- | --- | --- | --- | --- | --- | --- |
| ❑1 | ❑2 | ❑3 | ❑4 | ❑5 | ❑6 | ❑7 |

**DATI ANAGRAFICI E STATISTICI DELL’INTERVISTATO**

1. Lei è un uomo o una donna?
2. Uomo
3. Donna
4. Qual è il suo anno di nascita?|___|___|___|
5. Quale è la sua occupazione attuale?

❑1.Impiegato part-time

❑2.Impiegato full-time

❑3.Imprenditore/Libero professionista

❑4.Casalingo/a

❑5.Disoccupato

❑6.In cerca di occupazione

❑7.Pensionato

❑8.Altro (specificare):____________

1. (Se Q40=4-6) La sua condiziona lavorativa attuale è condizionata dal fatto di essere un caregiver di paziente affetto da patologia rara?

❑ Sì ❑ In parte sì ❑ Più no che sì ❑ No

*Ringraziare e chiudere*
