# Supplementary material for: Organizational and behavioral models in the management of patients with developmental and epileptic encephalopathy, Lennox-Gastaut syndrome and Dravet syndrome in Italy: a focus on the transition from pediatric to adult care
Source: Front Health Serv. 2025 Nov 7;5:1632564. doi: 10.3389/frhs.2025.1632564 (PMC12634588; doi:10.3389/frhs.2025.1632564)
Supplement: Supplementary file 3 [file Datasheet2.docx]

**Questionario**

Lei è un…

| 1. Neurologo dell’adulto | 🡪 continuare |
| --- | --- |
| 2. Neuropsichiatra infantile o Neurologo pediatra | 🡪 continuare |
| 3. Psichiatra | 🡪 CHIUDERE INTERVISTA |
| 4. Fisiatra | 🡪 CHIUDERE INTERVISTA |
| 5. Altro specialista | 🡪 CHIUDERE INTERVISTA |

1. Lei esercita in un reparto di…
2. Neurologia
3. Neurologia pediatrica
4. Neuropsichiatria infantile
5. Neurochirurgia
6. Neurofisiopatologia
7. Altro (specificare______________________)
8. Tra le seguenti patologie, di quale si occupa prevalentemente al momento? (Risposta singola)
   1. ❑ Sindrome di Lennox-Gastaut (SLG)
   2. ❑ Encefalopatia Epilettica e dello Sviluppo (DEE)
   3. ❑ Sindrome di Dravet (🡪 continua su variante questionario pg.7)
   4. ❑ Al momento non mi occupo di nessuna di queste patologie
9. (Se Q2=1) Attualmente segue pazienti con la Sindrome di Lennox-Gastaut (SLG)? Quanti?
10. ❑ Sì, seguo|__|__|__| (Se=0 🡪 chiudere l’intervista) pazienti con la Sindrome di Lennox-Gastaut (SLG)
11. ❑ Al momento non seguo alcun paziente con queste patologie 🡪CHIUDERE INTERVISTA
12. (Se Q2=2) Attualmente segue pazienti con Encefalopatia Epilettica e dello Sviluppo (DEE)? Quanti?
    1. ❑ Sì, seguo|__|__|__| (Se=0 🡪 chiudere l’intervista) pazienti con Encefalopatia Epilettica e dello Sviluppo (DEE)
    2. ❑ Al momento non seguo alcun paziente con questa patologia 🡪CHIUDERE INTERVISTA
13. Ha una competenza specifica nell’ambito del trattamento dell’Encefalopatia Epilettica e dello Sviluppo (DEE) o della Sindrome di Lennox-Gastaut (SLG)?

Risposta multipla (“no” è risposta esclusiva)

1. Sì, mi occupo del trattamento della DEE e/o SLG
2. Sì, Sono un referente del mio ospedale /a livello locale per questa patologia
3. Sì, Sono un referente a livello regionale per questa patologia
4. Sì, Sono un referente a livello nazionale per questa patologia
5. Sì, Sono un referente a livello internazionale per questa patologia
6. No 🡪CHIUDERE INTERVISTA
7. In che anno ha iniziato l’attività specialistica?

| ❑Prima del 1983 | 🡪CHIUDERE |
| --- | --- |
| Nel \|__\|__\|__\|__\| | 🡪 continuare |
| ❑Dopo il 2020 | 🡪CHIUDERE |

1. Qual è il suo Livello/posizione all’interno della struttura?
2. Direttore struttura complessa
3. Dirigente ad alta specializzazione
4. Dirigente di primo livello
5. Responsabile struttura semplice, dirigente di secondo livello

**DEE/LGS**

**FASE 1 – IDENTIFICAZIONE MODELLI ORGANIZZATIVI E PROCEDURE**

***Finalità:*** *Identificare organizzazione e procedure degli specialisti e dei centri di riferimento specializzati nel trattamento di DEE E LGS*

1. Parliamo dell’**attività della struttura** presso cui lei esercita. Sulla base della sua esperienza mediamente **in un anno**…

Q7a. Quanti pazienti con DEE/LGS si rivolgono alla sua struttura in un anno?

N° pazienti con DEE/LGS che si rivolgono alla mia struttura|__|__|__|(N° pazienti in valore assoluto; CHIUDERE SE 0)

*Di cui*
Q8a2. Con età 0>5 anni|__|__|__|(N° pazienti in valore assoluto; Q8a1 ≥ Q8a2)

Q8a3. Con età 6>14 anni|__|__|__|(N° pazienti in valore assoluto; Q8a1 ≥ Q8a3)

Q8a4. Con età 14>18 anni|__|__|__|(N° pazienti in valore assoluto; Q8a1 ≥ Q8a4)

Q8a5. Oltre 18 anni |__|__|__|(N° pazienti in valore assoluto; Q8a1 ≥ Q8a5)

1. Di questi (Q8a1) pazienti, quanti provengono da fuori Regione?

❑Nessuno

❑|__|__|__| provengono da altre Regioni rispetto a quella della mia struttura (Indicare le Regioni da cui provengono i pazienti ):_________________

1. Potrebbe dirmi quanti medici nel reparto/U.O. e quanti nel centro/ospedale in cui lavora, si occupano complessivamente della DEE o della LGS e quanti in particolare hanno competenze specifiche su questa patologia?

Q10A. |__|__|__| (N° medici che si occupano della DEE nel reparto/U.O.)

di cui con competenze specifiche su questa patologia|__|__|__|

Q10B. |__|__|__| (N° medici che si occupano della DEE nel centro/ospedale)

di cui con competenze specifiche su questa patologia|__|__|__|

Q10C. |__|__|__| (N° medici che si occupano della LGS nel reparto/U.O.)

di cui con competenze specifiche su questa patologia|__|__|__|

Q10D.|__|__|__| (N° medici che si occupano della LGS nel centro/ospedale)

di cui con competenze specifiche su questa patologia|__|__|__|

1. Nella sua struttura il paziente viene gestito….

❑ 1.Da un’equipe multidisciplinare

❑ 2.Dal singolo specialista, con il coinvolgimento di altri specialisti a seconda del caso

❑ 3. Altro: ________________________________________

1. (Se Q11= 1) Da quali specialisti è composta l’equipe? Si tratta di specialisti interni o esterni alla sua struttura?

| SPECIALISTA | Membro del team | Esterno |
| --- | --- | --- |
| 1. Neurologo | ❑ | ❑SI ❑NO |
| 1. Neuropsicologo infantile | ❑ | ❑SI ❑NO |
| 1. Neurofisiatra | ❑ | ❑SI ❑NO |
| 1. Neurologo pediatrico | ❑ | ❑SI ❑NO |
| 1. Neurochirurgo | ❑ | ❑SI ❑NO |
| 1. Psicologo | ❑ | ❑SI ❑NO |
| 1. Cardiologo | ❑ | ❑SI ❑NO |
| 1. Altro (specificare): | ❑ | ❑SI ❑NO |
| 1. Altro (specificare): | ❑ | ❑SI ❑NO |
| 1. Altro (specificare): | ❑ | ❑SI ❑NO |
| 1. Altro (specificare): | ❑ | ❑SI ❑NO |

**Q12a**. (Se Q11= 1) Come avviene la gestione in team del paziente con Sindrome di Dravet?

**Q12a1.** Ogni quanto avvengono le riunioni multidisciplinari? Ogni |__|__|__| ❑ giorni ❑ mesi

**Q12a2.** In quale modalità si svolgono le riunioni?

1. ❑ In presenza
2. ❑ Online
3. ❑ In entrambi i modi a seconda delle necessità

**Q12a3.** Chi è il responsabile della decisione terapeutica nel team?

| ❑ Neurologo |
| --- |
| ❑ Neuropsicologo infantile |
| ❑ Neurofisiatra |
| ❑ Neurologo pediatrico |
| ❑ Neurochirurgo |
| ❑ Psicologo |
| ❑ Cardiologo |
| ❑ Altro (specificare): |

1. (Se Q11= 2) Quali specialisti vengono solitamente coinvolti? Si tratta di specialisti interni o esterni alla struttura?

| SPECIALISTA | Membro del team | Esterno |
| --- | --- | --- |
| 1. Neurologo | ❑ | ❑SI ❑NO |
| 1. Neuropsicologo infantile | ❑ | ❑SI ❑NO |
| 1. Neurofisiatra | ❑ | ❑SI ❑NO |
| 1. Neurologo pediatrico | ❑ | ❑SI ❑NO |
| 1. Neurochirurgo | ❑ | ❑SI ❑NO |
| 1. Psicologo | ❑ | ❑SI ❑NO |
| 1. Cardiologo | ❑ | ❑SI ❑NO |
| 1. Altro (specificare): | ❑ | ❑SI ❑NO |
| 1. Altro (specificare): | ❑ | ❑SI ❑NO |
| 1. Altro (specificare): | ❑ | ❑SI ❑NO |
| 1. Altro (specificare): | ❑ | ❑SI ❑NO |

1. (Se Q11= 2) Quali sono le motivazioni che ostacolano la strutturazione di un team multidisciplinare per la gestione di questi pazienti?

❑ Motivi organizzativi/ logistici

❑ Alcuni specialisti non sono presenti nella struttura in cui lavoro

❑ Motivi economici

❑ Motivi politici

❑ Non è stato mai proposto

❑ Altro:_______________________________________

1. Relativamente alle **scelte terapeutiche**, quanto è d’accordo con le seguenti affermazioni?

*Risponda utilizzando una scala da 1 a 7 dove 1 equivale a completamente in disaccordo e 7 a completamente d’accordo.*

Una risposta per riga

|  | VALUTAZIONE |
| --- | --- |
| 1. Nel centro in cui lavoro prevale la tendenza a NON utilizzare farmaci innovativi | \|____\| |
| 1. Nel centro in cui lavoro per i medici le scelte terapeutiche circa i pazienti con LGS e DEE sono totalmente AUTONOME | \|____\| |
| 1. Nel centro in cui lavoro le scelte terapeutiche per i pazienti con LGS e DEE sono decise IN TEAM | \|____\| |
| 1. Nel centro in cui lavoro il PRONTUARIO FARMACEUTICO PONE LIMITI alle scelte terapeutiche per i pazienti con LGS e DEE | \|____\| |
| 1. Nel centro in cui lavoro il FATTORE COSTO impone dei limiti alle scelte terapeutiche per la LGS e DEE | \|____\| |
| 1. Nel centro in cui lavoro LE LINEE GUIDA TERAPEUTICHE interne alla struttura rappresentano un LIMITE alle scelte circa la LGS e le DEE | \|____\| |

1. Per i **pazienti che non possono recarsi facilmente al centro** per i controlli/follow-up o pazienti che possono seguire delle **cure domiciliari** avete previsto (sono possibili più risposte, risposta mod.6 esclusiva):

1. un training di formazione per la gestione della patologia/terapie

2. un sistema di monitoraggio a distanza

3. un sistema di telemedicina/teleassistenza

4. dei patient support program

5. Altro specificare____________

6. nessun servizio/attività

1. È previsto un servizio di **assistenza psicologica** per questi pazienti?

1. Sì, interno al mio reparto

2. Sì, interno al centro presso cui lavoro

3. Sì, ma come consulenza esterna/a chiamata

4. No

5. Altro specificare____________

1. È previsto un servizio di **assistenza psicologica** per le **famiglie** dei pazienti?

1. Sì, interno al mio reparto

2. Sì, interno al centro presso cui lavoro

3. Sì, ma come consulenza esterna/a chiamata

4. No

5. Altro specificare____________

**FASE 2 – RICOSTRUZIONE DEL PATIENT JOURNEY**

***Finalità:***  *Tracciare il percorso dal momento in cui si sono presentati i primi sintomi, identificando gli snodi e i momenti significativi*

1. In media dopo quanto tempo dalla comparsa dei primi sintomi si arriva ad una diagnosi di LGS e DEE?
   Solitamente la diagnosi arriva dopo |__|__|__| mesi dalla comparsa dei primi sintomi
2. Pensando alla storia dei suoi pazienti, è frequente il verificarsi di ritardi nella diagnosi di queste patologie?
3. Sì, sia per la LGS che per le DEE
4. Sì, ma soltanto nel caso di ❑ LGS ❑DEE
5. Raramente
6. No

**Q19a.** (Se Q19=1) Quali sono le conseguenze di tale ritardo diagnostico sul percorso del paziente?

______________________________________________________________________________________________________________________________________________________­­­­­­­­­­__________________________________________

1. Quali esami vengono condotti su un paziente per il quale si sospetta la LGS o DEE?

| **ESAMI DIAGNOSTICI** | **LGS** | **DEE** |
| --- | --- | --- |
| 1. EEG (veglia) | ❑ | ❑ |
| 1. EEG (veglia e sonno) | ❑ | ❑ |
| 1. RM cervello | ❑ | ❑ |
| 1. Esame obiettivo | ❑ | ❑ |
| 1. Esami di laboratorio | ❑ | ❑ |
| 1. Risonanza magnetica nucleare (RMN) | ❑ | ❑ |
| 1. TAC | ❑ | ❑ |
| 1. Altro (Specificare):_____________ | ❑ | ❑ |

1. Quali specialisti sono coinvolti nel percorso diagnostico della LGS e delle DEE?

❑ Neurologo

❑ Neuropsichiatra

❑ Fisiatra

❑ Neurofisiatra

❑ Psicologo

❑ Altro (specificare):____________

1. Quale specialista comunica solitamente la diagnosi al paziente e alla famiglia?

❑ Neurologo

❑ Neuropsichiatra

❑ Fisiatra

❑ Neurofisiatra

❑ Altro (specificare):____________

**Q22a.** wÈ presente lo psicologo durante la comunicazione della diagnosi? ❑ Sì ❑ No

1. Considerando le principali **terapie farmacologiche** di seguito indicate qual è la quota di pazienti con LGS e DEE a cui vengono prescritte.

| **TERAPIE FARMACOLOGICHE** | **% PAZIENTI CON LGS** | **% PAZIENTI CON DEE** |
| --- | --- | --- |
| ❑Felbamato | \|__\|__\|__\| % | \|__\|__\|__\| % |
| ❑Lamotrigina | \|__\|__\|__\| % | \|__\|__\|__\| % |
| ❑Topiramato | \|__\|__\|__\| % | \|__\|__\|__\| % |
| ❑Levetiracetam | \|__\|__\|__\| % | \|__\|__\|__\| % |
| ❑Rufinamide | \|__\|__\|__\| % | \|__\|__\|__\| % |
| ❑ Acido valproico | \|__\|__\|__\| % | \|__\|__\|__\| % |
| ❑ Fenfluramina | \|__\|__\|__\| % | \|__\|__\|__\| % |
| ❑ Fenobarbitale | \|__\|__\|__\| % | \|__\|__\|__\| % |
| ❑ Progabide | \|__\|__\|__\| % | \|__\|__\|__\| % |
| ❑ Gabapentin | \|__\|__\|__\| % | \|__\|__\|__\| % |
| ❑ Clobazam | \|__\|__\|__\| % | \|__\|__\|__\| % |
| ❑ Carbamazepina | \|__\|__\|__\| % | \|__\|__\|__\| % |
| ❑ Fenitoina | \|__\|__\|__\| % | \|__\|__\|__\| % |
| ❑ Cannabidiolo | \|__\|__\|__\| % | \|__\|__\|__\| % |
| ❑ Altro (Specificare):_____________ | \|__\|__\|__\| % | \|__\|__\|__\| % |

1. Le terapie indicate (Q12) vengono prescritte…

❑In monoterapia

❑In associazione

Se in associazione: Indicare le associazioni più frequenti (sono possibili più risposte):

1)_________________
2)_________________
3)_________________

1. Quali altri **trattamenti e terapie** sono Solitamente seguiti dai pazienti con LGS e DEE? (sono possibili più risposte)
2. ❑Logopedista
3. ❑Psicologo
4. ❑Fisioterapista
5. ❑Cardiologo
6. ❑Fisiatra
7. ❑Nutrizionista
8. ❑Educatore comportamentale
9. ❑Altro (specificare):____________
10. Con che frequenza in numero di mesi avvengono in media le **visite di controllo** (Q7a1) per i pazienti con LGS/DEE gestiti e trattati nella sua struttura?

In media ogni |___| mesi.

1. Facendo 100 i suoi pazienti LGS/ DEE che vengono gestiti e trattati nella sua struttura, dopo la prima prescrizione in che modalità vengono seguiti nelle **visite di controllo**? La somma delle % deve fare 100

|__|__|__| %in presenza nella mia struttura

|__|__|__| % in presenza in un'altra struttura (specificare________)

|__|__|__| % in presenza a domicilio

|__|__|__| % online con piattaforma di telemedicina dedicata(specificare___)

|__|__|__| % in videochiamata (es. WhatsApp, Zoom…)

|__|__|__| % telefonicamente

|__|__|__| % Altro specificare ______________

**FASE 3 – TRANSIZIONE ALL’ETA’ ADULTA**

***Finalità:***  *Evidenziare i modelli assistenziali nella gestione del passaggio dall’età pediatrica all’età adulta*

1. Nella sua struttura attualmente esiste un **percorso di transizione strutturato**, che seguendo procedure e tempistiche stabilite e fisse conduca i pazienti con LGS e DEE dal Centro pediatrico al Centro dell’adulto?

|  | **LGS** | **DEE** |
| --- | --- | --- |
| 1. Attualmente no | ❑ | ❑ |
| 2. Al momento no, ma lo stiamo strutturando | ❑ | ❑ |
| 3. Esiste e viene applicato per tutti i pazienti | ❑ | ❑ |
| 4. Esiste un percorso di transizione, ma è molto variabile e non viene applicato per tutti i pazienti | ❑ | ❑ |

1. In base alla sua esperienza, a quale età avviene il passaggio dal centro pediatrico al centro dell’adulto per i pazienti con LGS e DEE?

**Q29a.Sindrome Lennox-Gastaut**

Il |__|__|__| % dei pazienti con LGS passa al centro dell’adulto a 18 anni

Il |__|__|__| % dei pazienti con LGS passa al centro dell’adulto entro i 20 anni

Il |__|__|__| % dei pazienti con LGS passa al centro dell’adulto entro i 25 anni

Il |__|__|__| % dei pazienti con LGS passa al centro dell’adulto oltre i 25 anni

Il |__|__|__| % dei pazienti con LGS non effettua questo passaggio

**Q29b. Encefalopatia epilettica e dello sviluppo**

Il |__|__|__| % dei pazienti con DEE passa al centro dell’adulto a 18 anni

Il |__|__|__| % dei pazienti con DEE passa al centro dell’adulto entro i 20 anni

Il |__|__|__| % dei pazienti con DEE passa al centro dell’adulto entro i 25 anni

Il |__|__|__| % dei pazienti con DEE passa al centro dell’adulto oltre i 25 anni

Il |__|__|__| % dei pazienti con DEE non effettua questo passaggio

1. (Se Q28=2-4) Da quanti anni esiste questo percorso di transizione? Da |__|__| anni
2. (Se Q28=2-4) In quali step si articola il percorso di transizione verso il centro dell’adulto?
3. ____________________
4. ____________________
5. ____________________
6. ____________________
7. ____________________
8. ____________________
9. ____________________

**Q31a1.** È previsto il coinvolgimento del medico di base? ❑ Sì ❑No ❑ Non so

**Q31a2.** Quando dura complessivamente il periodo di passaggio? |__|__|__| ❑ giorni ❑ mesi

1. I pazienti e le famiglie di solito….

❑1. Accettano di buon grado la transizione al centro dell’adulto

❑ 2. Sono all’inizio restii alla transizione, ma poi la accettano di buon grado

❑ 3. Mostrano forti resistenze alla transizione al centro dell’adulto

❑ 4. Non accettano la transizione al centro dell’adulto e richiedono la permanenza nel centro pediatrico

**Q33b**. Se (Q32= 2,3,4) Quali sono i motivi di questa resistenza?

__________________________________________________________________________________________________________________________________________________________________________________

1. In base alla sua esperienza, quale è la percentuale di pazienti che smette di frequentare il Centro dell’adulto una volta effettuato il passaggio?

Il **Q33a.**|__|__|__| % dei pazienti non frequentano più il centro dopo **Q33b.** |__|__|__| anno/i dalla presa in carico.

**Q33c**. Come mai secondo lei il (Q33a) non frequenta più il centro una volta diventati adulti? ____________________________________________________________________________________________________________________________________________________________________________

1. In base alla sua esperienza, i pazienti che abbandonano il centro dell’adulto, come vengono gestiti?
2. Domiciliarmente, con la ASL territoriale
3. Domiciliarmente, da uno specialista privato
4. Cambiano struttura e si recano (specificare tipo di centro di destinazione):_______________
5. Altro (specificare):___________________
6. Solitamente quali **trattamenti e terapie** vengono proseguiti dal paziente con DEE/LGS in età adulta? (sono possibili più risposte)

❑Logopedista

❑Psicologo

❑Fisioterapista

❑Cardiologo

❑Fisiatra

❑Nutrizionista

❑Educatore comportamentale

❑Altro (specificare):____________

1. Quali sono **le prime tre criticità che incontra, in quanto medico**, nella gestione dei pazienti con LGS/ DEE?

1° |_____________________________|

2° |_____________________________|

3° |_____________________________|

1. Quali sono **le prime tre criticità che le vengono riferite**  dai pazienti e dalle famiglie durante il percorso di cura?

1° |_____________________________|

2° |_____________________________|

3° |_____________________________|

**DATI ANAGRAFICI E STATISTICI DELL’INTERVISTATO**

1. Lei è un uomo o una donna?
2. Uomo
3. Donna
4. Qual è il suo anno di nascita?|___|___|___|
5. In quale centro esercita la Sua attività professionale?
6. In quale regione esercita la Sua attività professionale?
7. In quale comune esercita la Sua attività professionale?
8. Tipo di ospedale/centro in cui lei lavora

- Azienda Ospedaliera
- Azienda Ospedaliera Universitaria
- Presidi Ospedalieri Territoriali (ASP/ASL/AUSL)
- Case di Cura (Privato Accreditato)
- A Gestione diretta (Sperimentazioni Gestionali Pubblico-Privato)
- IRCSS
- Altro (specificare___)

*Ringraziare e chiudere*

**SINDROME DI DRAVET**

1. (Se Q2=2) Attualmente segue pazienti Sindrome di Dravet? Quanti?
   1. ❑ Sì, seguo|__|__|__| (Se=0 🡪 chiudere l’intervista) pazienti con Sindrome di Dravet
   2. ❑ Al momento non seguo alcun paziente con questa patologia 🡪CHIUDERE INTERVISTA
2. Ha una competenza specifica nell’ambito del trattamento della Sindrome di Dravet?

Risposta multipla (“no” è risposta esclusiva)

1. Sì, mi occupo del trattamento della Sindrome di Dravet
2. Sì, Sono un referente del mio ospedale /a livello locale per questa patologia
3. Sì, Sono un referente a livello regionale per questa patologia
4. Sì, Sono un referente a livello nazionale per questa patologia
5. Sì, Sono un referente a livello internazionale per questa patologia
6. No 🡪CHIUDERE INTERVISTA
7. In che anno ha iniziato l’attività specialistica?

| ❑Prima del 1983 | 🡪CHIUDERE |
| --- | --- |
| Nel \|__\|__\|__\|__\| | 🡪 continuare |
| ❑Dopo il 2020 | 🡪CHIUDERE |

1. Qual è il suo Livello/posizione all’interno della struttura?
2. Direttore struttura complessa
3. Dirigente ad alta specializzazione
4. Dirigente di primo livello
5. Responsabile struttura semplice, dirigente di secondo livello

**DEE/LGS**

**FASE 1 – IDENTIFICAZIONE MODELLI ORGANIZZATIVI E PROCEDURE**

***Finalità:*** *Identificare organizzazione e procedure degli specialisti e dei centri di riferimento specializzati nel trattamento di DEE E LGS*

1. Parliamo dell’**attività della struttura** presso cui lei esercita. Sulla base della sua esperienza mediamente **in un anno**…

Q5a1. Quanti pazienti con Sindrome di Dravet si rivolgono alla sua struttura in un anno?

N° pazienti con Sindrome di Dravet che si rivolgono alla mia struttura|__|__|__|(N° pazienti in valore assoluto; CHIUDERE SE 0)

*Di cui*
55a2. Con età 0>5 anni|__|__|__|(N° pazienti in valore assoluto; Q5a1 ≥ Q5a2)

Q5a3. Con età 6>14 anni|__|__|__|(N° pazienti in valore assoluto; Q5a1 ≥ Q5a3)

Q5a4. Con età 14>18 anni|__|__|__|(N° pazienti in valore assoluto; Q5a1 ≥ Q5a4)

Q5a5. Oltre 18 anni |__|__|__|(N° pazienti in valore assoluto; Q8a1 ≥ Q5a5)

1. Di questi (Q5a1) pazienti, quanti provengono da fuori Regione?

❑Nessuno

❑|__|__|__| provengono da altre Regioni rispetto a quella della mia struttura (Indicare le Regioni da cui provengono i pazienti ):_________________

1. Potrebbe dirmi quanti medici nel reparto/U.O. e quanti nel centro/ospedale in cui lavora, si occupano complessivamente della Sindrome di Dravet e quanti in particolare hanno competenze specifiche su questa patologia?

Q7a1. |__|__|__| (N° medici che si occupano della Sindrome di Dravet nel reparto/U.O.)

di cui con competenze specifiche su questa patologia|__|__|__|

Q7a2. |__|__|__| (N° medici che si occupano della Sindrome di Dravet nel centro/ospedale)

di cui con competenze specifiche su questa patologia|__|__|__|

1. Nella sua struttura il paziente viene gestito….

❑ 1.Da un’equipe multidisciplinare

❑ 2.Dal singolo specialista, con il coinvolgimento di altri specialisti a seconda del caso

❑ 3. Altro: ________________________________________

1. (Se Q10= 1) Da quali specialisti è composta l’equipe? Si tratta di specialisti interni o esterni alla sua struttura?

| SPECIALISTA | Membro del team | Esterno |
| --- | --- | --- |
| 1. Neurologo | ❑ | ❑SI ❑NO |
| 1. Neuropsicologo infantile | ❑ | ❑SI ❑NO |
| 1. Neurofisiatra | ❑ | ❑SI ❑NO |
| 1. Neurologo pediatrico | ❑ | ❑SI ❑NO |
| 1. Neurochirurgo | ❑ | ❑SI ❑NO |
| 1. Psicologo | ❑ | ❑SI ❑NO |
| 1. Cardiologo | ❑ | ❑SI ❑NO |
| 1. Altro (specificare): | ❑ | ❑SI ❑NO |
| 1. Altro (specificare): | ❑ | ❑SI ❑NO |
| 1. Altro (specificare): | ❑ | ❑SI ❑NO |
| 1. Altro (specificare): | ❑ | ❑SI ❑NO |

1. (Se Q10= 1) Come avviene la gestione in team del paziente con Sindrome di Dravet?

**Q10a1.** Ogni quanto avvengono le riunioni multidisciplinari? Ogni |__|__|__| ❑ giorni ❑ mesi

**Q10a2.** In quale modalità si svolgono le riunioni?

1. ❑ In presenza
2. ❑ Online
3. ❑ In entrambi i modi a seconda delle necessità

**Q10a3.** Chi è il responsabile della decisione terapeutica nel team?

| ❑ Neurologo |
| --- |
| ❑ Neuropsicologo infantile |
| ❑ Neurofisiatra |
| ❑ Neurologo pediatrico |
| ❑ Neurochirurgo |
| ❑ Psicologo |
| ❑ Cardiologo |
| ❑ Altro (specificare): |

1. (Se Q10= 2) Quali specialisti vengono solitamente coinvolti? Si tratta di specialisti interni o esterni alla struttura?

| SPECIALISTA | Membro del team | Esterno |
| --- | --- | --- |
| 1. Neurologo | ❑ | ❑SI ❑NO |
| 1. Neuropsicologo infantile | ❑ | ❑SI ❑NO |
| 1. Neurofisiatra | ❑ | ❑SI ❑NO |
| 1. Neurologo pediatrico | ❑ | ❑SI ❑NO |
| 1. Neurochirurgo | ❑ | ❑SI ❑NO |
| 1. Psicologo | ❑ | ❑SI ❑NO |
| 1. Cardiologo | ❑ | ❑SI ❑NO |
| 1. Altro (specificare): | ❑ | ❑SI ❑NO |
| 1. Altro (specificare): | ❑ | ❑SI ❑NO |
| 1. Altro (specificare): | ❑ | ❑SI ❑NO |
| 1. Altro (specificare): | ❑ | ❑SI ❑NO |

1. (Se Q10= 2) Quali sono le motivazioni che ostacolano la strutturazione di un team multidisciplinare per la gestione di questi pazienti?

❑ Motivi organizzativi/ logistici

❑ Alcuni specialisti non sono presenti nella struttura in cui lavoro

❑ Motivi economici

❑ Motivi politici

❑ Non è stato mai proposto

❑ Altro:_______________________________________

1. Relativamente alle **scelte terapeutiche**, quanto è d’accordo con le seguenti affermazioni?

*Risponda utilizzando una scala da 1 a 7 dove 1 equivale a completamente in disaccordo e 7 a completamente d’accordo.*

Una risposta per riga

|  | VALUTAZIONE |
| --- | --- |
| 1. Nel centro in cui lavoro prevale la tendenza a NON utilizzare farmaci innovativi | \|____\| |
| 1. Nel centro in cui lavoro per i medici le scelte terapeutiche circa i pazienti con Sindrome di Dravet sono totalmente AUTONOME | \|____\| |
| 1. Nel centro in cui lavoro le scelte terapeutiche per i pazienti con Sindrome di Dravet sono decise IN TEAM | \|____\| |
| 1. Nel centro in cui lavoro il PRONTUARIO FARMACEUTICO PONE LIMITI alle scelte terapeutiche per i pazienti con Sindrome di Dravet | \|____\| |
| 1. Nel centro in cui lavoro il FATTORE COSTO impone dei limiti alle scelte terapeutiche per la Sindrome di Dravet | \|____\| |
| 1. Nel centro in cui lavoro LE LINEE GUIDA TERAPEUTICHE interne alla struttura rappresentano un LIMITE alle scelte circa la Sindrome di Dravet | \|____\| |

1. Per i **pazienti che non possono recarsi facilmente al centro** per i controlli/follow-up o pazienti che possono seguire delle **cure domiciliari** avete previsto (sono possibili più risposte, risposta mod.6 esclusiva):

1. un training di formazione per la gestione della patologia/terapie

2. un sistema di monitoraggio a distanza

3. un sistema di telemedicina/teleassistenza

4. dei patient support program

5. Altro specificare____________

6. nessun servizio/attività

1. È previsto un servizio di **assistenza psicologica** per questi pazienti?

1. Sì, interno al mio reparto

2. Sì, interno al centro presso cui lavoro

3. Sì, ma come consulenza esterna/a chiamata

4. No

5. Altro specificare____________

1. È previsto un servizio di **assistenza psicologica** per le **famiglie** dei pazienti?

1. Sì, interno al mio reparto

2. Sì, interno al centro presso cui lavoro

3. Sì, ma come consulenza esterna/a chiamata

4. No

5. Altro specificare____________

**FASE 2 – RICOSTRUZIONE DEL PATIENT JOURNEY**

***Finalità:***  *Tracciare il percorso dal momento in cui si sono presentati i primi sintomi, identificando gli snodi e i momenti significativi*

1. In media dopo quanto tempo dalla comparsa dei primi sintomi si arriva ad una diagnosi di LGS e DEE?
   Solitamente la diagnosi arriva dopo |__|__|__| mesi dalla comparsa dei primi sintomi
2. Pensando alla storia dei suoi pazienti, è frequente il verificarsi di ritardi nella diagnosi per questa patologia?
3. Sì, è frequente
4. Qualche volta
5. Raramente
6. No

**Q14a1.** (Se Q=14) Quali sono le conseguenze di tale ritardo diagnostico sul percorso del paziente?

______________________________________________________________________________________________________________________________________________________­­­­­­­­­­__________________________________________

1. Quali esami vengono condotti su un paziente per il quale si sospetta la Sindrome di Dravet?

| 1. EEG (veglia) | ❑ | ❑ |
| --- | --- | --- |
| 1. EEG (veglia e sonno) | ❑ | ❑ |
| 1. RM cervello | ❑ | ❑ |
| 1. Esame obiettivo | ❑ | ❑ |
| 1. Esami di laboratorio | ❑ | ❑ |
| 1. Risonanza magnetica nucleare (RMN) | ❑ | ❑ |
| 1. Pannelli genetici | ❑ | ❑ |
| 1. Cariotipo | ❑ | ❑ |
| 1. Array CGH | ❑ | ❑ |
| 1. TAC | ❑ | ❑ |
| 1. Altro (Specificare):_____________ | ❑ | ❑ |

1. Quali specialisti sono coinvolti nel percorso diagnostico della Sindrome di Dravet?

❑ Neurologo

❑ Neuropsichiatra

❑ Fisiatra

❑ Neurofisiatra

❑ Psicologo

❑ Genetista

❑ Altro (specificare):____________

1. Quale specialista comunica solitamente la diagnosi al paziente e alla famiglia?

❑ Neurologo

❑ Neuropsichiatra

❑ Fisiatra

❑ Neurofisiatra

❑ Altro (specificare):____________

1. È presente lo psicologo durante la comunicazione della diagnosi? ❑ Sì ❑ No
2. Considerando le principali **terapie farmacologiche** di seguito indicate qual è la quota di pazienti con Sindrome di Dravet a cui vengono prescritte.

| **TERAPIE FARMACOLOGICHE** | **% PAZIENTI CON LGS** | **% PAZIENTI CON DEE** |
| --- | --- | --- |
| ❑ Valproato | \|__\|__\|__\| % | \|__\|__\|__\| % |
| ❑ Clobazam | \|__\|__\|__\| % | \|__\|__\|__\| % |
| ❑ Fenfluramina | \|__\|__\|__\| % | \|__\|__\|__\| % |
| ❑ Stiripentolo | \|__\|__\|__\| % | \|__\|__\|__\| % |
| ❑ Clonazepam | \|__\|__\|__\| % | \|__\|__\|__\| % |
| ❑ Midazolam | \|__\|__\|__\| % | \|__\|__\|__\| % |
| ❑ Fenfluramina | \|__\|__\|__\| % | \|__\|__\|__\| % |
| ❑ Cannabidiolo | \|__\|__\|__\| % | \|__\|__\|__\| % |
| ❑ Altro (Specificare):_____________ | \|__\|__\|__\| % | \|__\|__\|__\| % |

1. Le terapie indicate (Q12) vengono prescritte…

❑In monoterapia

❑In associazione

Se in associazione: Indicare le associazioni più frequenti (sono possibili più risposte):

1)_________________
2)_________________
3)_________________

1. Quali altri **trattamenti e terapie** sono Solitamente seguiti dai pazienti con Sindrome di Dravet? (sono possibili più risposte)
2. ❑Logopedista
3. ❑Psicologo
4. ❑Fisioterapista
5. ❑Cardiologo
6. ❑Fisiatra
7. ❑Nutrizionista
8. ❑Educatore comportamentale
9. ❑Altro (specificare):____________
10. Con che frequenza in numero di mesi avvengono in media le **visite di controllo** (Q7a1) per i pazienti con Sindrome di Dravet gestiti e trattati nella sua struttura?

In media ogni |___| mesi.

1. Facendo 100 i suoi pazienti con Sindrome di Dravet che vengono gestiti e trattati nella sua struttura, dopo la prima prescrizione in che modalità vengono seguiti nelle **visite di controllo**? La somma delle % deve fare 100

|__|__|__| %in presenza nella mia struttura

|__|__|__| % in presenza in un'altra struttura (specificare________)

|__|__|__| % in presenza a domicilio

|__|__|__| % online con piattaforma di telemedicina dedicata(specificare___)

|__|__|__| % in videochiamata (es. WhatsApp, Zoom…)

|__|__|__| % telefonicamente

|__|__|__| % Altro specificare ______________

**FASE 3 – TRANSIZIONE ALL’ETA’ ADULTA**

***Finalità:***  *Evidenziare i modelli assistenziali nella gestione del passaggio dall’età pediatrica all’età adulta*

1. Nella sua struttura attualmente esiste un **percorso di transizione strutturato**, che seguendo procedure e tempistiche stabilite e fisse conduca i pazienti con Sindrome di Dravet dal Centro pediatrico al Centro dell’adulto?

❑1. Attualmente no

❑2. Al momento no, ma lo stiamo strutturando

❑3. Esiste e viene applicato per tutti i pazienti

❑4. Esiste un percorso di transizione, ma è molto variabile e non viene applicato per tutti i pazienti

1. In base alla sua esperienza, a quale età avviene il passaggio dal centro pediatrico al centro dell’adulto per i pazienti con Sindrome di Dravet?

Il |__|__|__| % dei pazienti con SD passa al centro dell’adulto a 18 anni

Il |__|__|__| % dei pazienti con SD passa al centro dell’adulto entro i 20 anni

Il |__|__|__| % dei pazienti con SD passa al centro dell’adulto entro i 25 anni

Il |__|__|__| % dei pazienti con SD passa al centro dell’adulto oltre i 25 anni

Il |__|__|__| % dei pazienti con SD non effettua questo passaggio

1. (Se Q24=2-4) Da quanti anni esiste questo percorso di transizione? Da |__|__| anni
2. (Se Q24=2-4) In quali step si articola il percorso di transizione verso il centro dell’adulto?
3. ____________________
4. ____________________
5. ____________________
6. ____________________
7. ____________________
8. ____________________
9. ____________________

**Q27a1.** È previsto il coinvolgimento del medico di base? ❑ Sì ❑No ❑ Non so

**Q27a2.** Quando dura complessivamente il periodo di passaggio? |__|__|__| ❑ giorni ❑ mesi

1. In base alla sua esperienza, quale è la percentuale di pazienti che smette di frequentare il Centro dell’adulto una volta effettuato il passaggio?

Il **Q28a.**|__|__|__| % dei pazienti non frequentano più il centro dopo **Q28b.** |__|__|__| anno/i dalla presa in carico.

**Q28b**. Come mai secondo lei il (Q28a) non frequenta più il centro una volta diventati adulti? ____________________________________________________________________________________________________________________________________________________________________________

1. In base alla sua esperienza, i pazienti che abbandonano il centro dell’adulto, come vengono gestiti?
2. Domiciliarmente, con la ASL territoriale
3. Domiciliarmente, da uno specialista privato
4. Cambiano struttura e si recano (specificare tipo di centro di destinazione):_______________
5. Altro (specificare):___________________
6. Quali sono **le prime tre criticità che incontra, in quanto medico**, nella gestione dei pazienti con Sindrome di Dravet?

1° |_____________________________|

2° |_____________________________|

3° |_____________________________|

1. Quali sono **le prime tre criticità che le vengono riferite**  dai pazienti e dalle famiglie durante il percorso di cura?

1° |_____________________________|

2° |_____________________________|

3° |_____________________________|
